# Glioblastoma cytotoxicity conferred through dual disruption of endolysosomal homeostasis by Vacquinol-1

**DOI:** 10.1093/noajnl/vdab152

**Published:** 2021-10-15

**Authors:** Dongoh Kwak, Lars G J Hammarström, Martin Haraldsson, Patrik Ernfors

**Affiliations:** 1 Division of Molecular Neurobiology, Department of Medical Biochemistry and Biophysics, Karolinska Institutet, Stockholm, Sweden; 2 Chemical Biology Consortium Sweden (CBCS), Science for Life Laboratory, Department of Medical Biochemistry and Biophysics, Karolinska Institutet, Stockholm, Sweden

**Keywords:** cationic amphiphilic drug, endolysosome, glioblastoma, nonapoptotic cell death

## Abstract

**Background:**

Increased membrane trafficking is observed in numerous cancer types, including glioblastoma. Targeting the oncogenic driven acquired alterations in membrane trafficking by synthetic cationic amphiphilic small molecules has recently been shown to induce death of glioblastoma cells, although the molecular targets are unknown.

**Methods:**

The mechanism of action of the cationic amphiphilic drug Vacquinol-1 (Vacq1)-induced cytotoxicity was investigated using cell biology, biochemistry, functional experiments, chemical biology, unbiased antibody-based post-translation modification profiling, and mass spectrometry-based chemical proteomic analysis on patient-derived glioblastoma cells.

**Results:**

Vacq1 induced two types of abnormal endolysosomal vesicles, enlarged vacuoles and acidic vesicle organelles (AVOs). Mechanistically, enlarged vacuoles were formed by the impairment of lysosome reformation through the direct interaction and inhibition of calmodulin (CaM) by Vacq1, while AVO formation was induced by Vacq1 accumulation and acidification in the endosomal compartments through its activation of the v-ATPase. As a consequence of v-ATPase activation, cellular ATP consumption markedly increased, causing cellular energy shortage and cytotoxicity. This effect of Vacq1 was exacerbated by its inhibitory effects on calmodulin, causing lysosomal depletion and a failure of acidic vesicle organelle clearance.

**Conclusion:**

Our study identifies the targets of Vacq1 and the mechanisms underlying its selective cytotoxicity in glioblastoma cells. The dual function of Vacq1 sets in motion a glioblastoma-specific vicious cycle of ATP consumption resulting in cellular energy crisis and cell death.

**Key Points**

- Molecular mechanisms of cationic amphiphilic Vacquinol-1 cytotoxicity by targeting endolysosome homeostasis.
- Therapeutic utility of simultaneous interference at multiple sites of the endolysosome pathway for glioblastoma.

**Importance of the Study**

These results show that the glioblastoma cytotoxicity of Vacq1 is conferred through specific interactions within the endolysosomal compartment. Identification of the targets and mechanism of action of Vacq1 advances our understanding of the vacuolization phenotype observed in cancer cells and opens for the development of more specific candidate drugs with robust *in vivo* efficacy.

Glioblastoma is notoriously difficult to treat. Even though recent strategies identifying genetic, epigenetic, and intracellular gene expression programs defining glioblastoma subtypes have markedly advanced knowledge on the pathophysiology, there has been a lack of progress in improving the survival of patients and it remains an incurable disease.^1^ Alteration in the genomic landscape of glioblastoma^2^ leads to metabolic fueling, increased cell proliferation, and enhanced survival and invasion properties, while preventing tumor cells from apoptosis and activation of cell cycle checkpoints. However, glioblastoma is characterized by transcriptional and genetic intertumoral heterogeneity^3,4^ as well as intratumoral heterogeneity.^5,6^ Hence, the cellular complexity underlying tumor propagation is highly complex within patients that may explain why targeting any individual protein or particular cell type has not been successful in the clinic. Thus, targeting particular pathways with aberrant activity due to genetic alterations may lead to expansion of cells with other genetic profiles within the tumor and driving a positive selection for evolution of the genetically unstable tumor cells, both leading to tumor maintenance and relapse.^7^ Although the concept of precision cancer medicine has transformed the clinical practice of many cancer treatment, the lack of clinically actionable mutations in glioblastoma warrants alternative approaches and expanding the scope of drugging strategies. Because the mutational landscape rewires cellular physiology,^8^ identifying context-specific vulnerabilities by screening for sensitivity of cancer cells to small molecules is one such approach.^9,10^ Recent reports indicate macropinocytosis and membrane trafficking in vacuoles and in the endolysosomal pathway as a pervasive feature of many tumor types,^11^ including glioblastoma.^12,13^

Increased membrane trafficking and lysosomal activation in cancer promote tumor cell metabolism and aggressiveness,^14^ however, it may also sensitize tumor cells to cell death. Such acquired vulnerability can be targeted by cationic amphiphilic drugs (CAD) that induces membrane trafficking and vacuolization, resulting in a rapid nonapoptotic death in several types of cancer.^15,16^ The CADs comprise a broad spectrum of compound classes with the common feature of an amphiphilic nature brought about by a hydrophobic ring structure and a hydrophilic side chain containing cationic amine groups. The CAD, Vacq1 has potent antitumor effects by inducing macropinocytosis followed by massive vacuolization and nonapoptotic cell death.^13,17–20^ However, although potent at lower micromolar concentration *in vitro* resulting in complete cell death, it has only limited efficacy *in vivo* in syngeneic glioblastoma models at the highest tolerated doses.^19^ Thus, Vacq1 has nonspecific or unrelated toxicity at high doses limiting its clinical utility as a monotherapy. Understanding the mechanism of action of Vacq1 could provide insights into glioblastoma pathophysiology and provide a basis for developing new compounds with higher tumor-selective efficacy and reduced general toxicity. Here, we report a detailed analysis of the molecular mechanisms of Vacq1. We find two main mechanisms accounting for the tumor-specific toxicity of Vacq1; activation of vacuolar ATPase in acidic vesicle organelles (AVOs) resulting in metabolic catastrophe due to ATP depletion and an interaction with calmodulin (CaM) in enlarged lysosomal vacuoles perturbing membrane fusion and intracellular vesicular trafficking.

## Materials and Methods

### Cell Culture

Patient-derived glioblastoma cells (U3013MG, U3024MG)^21^ were grown in Neurocult NS-A basal medium (Human) (STEMCELL Technologies Inc.) supplemented with N2 (Invitrogen), B27 (Invtrogen), 10 ng/ml EGF (Peprotech), and 10 ng/ml bFGF (Peprotech). Cell culture dishes were precoated with 10 µg/ml laminin (Sigma-Aldrich) for 3 h. Normal human foreskin fibroblast cells (CCD-1112Sk) were obtained from ATCC and maintained in DMEM medium (Invitrogen) supplemented with 10% FBS (Invitrogen). Normal human astrocytes (NHA) were purchased from Lonza and cultured according to the manufacturer’s protocol.

### Cell Viability Assay

Three thousand cells/well were seeded in 96 well plates. Cell viability was assessed using CellTiter-Glo (Promega G7571) or CytoTox-Glo (Promega G9290) 48 h post-treatment with different reagents as indicated in the corresponding figure legends. Unless otherwise specified, all analyses were performed at indicated concentrations in a volume of 80 µl. Luminescence was measured on a FLUOstar Omega microplate reader (BMG Labtech). Viability was calculated as the percentage of control (DMSO treated cells) with at least three replicates for each concentration. IC_50_ was determined as the concentration corresponding half-maximal growth inhibition.

### Microscopic Analysis

U3013MG, U3024MG, or fibroblast cells were plated laminin-coated chambered cell culture slide. Cells were imaged on Olympus FV1000 confocal microscope or Olympus IX73 widefield microscope. For organelle labeling, GFP-tagged CellLight® Reagent BacMam 2.0 (Molecular Probes, see Supplementary Methods for reagent list) were added to the culture in a final concentration of 30 particles per cell, according to the manufacturer’s protocol. After 24 h, cells were briefly rinsed and treated with Vacq1. For pulse-chase monitoring of plasma membrane, cells were pulsed with 5 µg/ml Alexa Fluor 350-WGA (Thermo Fisher Scientific, #W11263) for 30 min, rinsed, and treated with Vacq1. For AVO labeling, cells were incubated in 100 nM of Lysotracker Red DND-99 (Thermo Fisher Scientific, #L7528) for 5 min in the incubator to prior to imaging. Hoechst 33342 (Thermo Fisher Scientific, #H21492) was used for labeling DNA at 1 µg/ml. Vacq1-Click was labeled by Alexa Fluor 555 Azide (Thermo Fisher Scientific, #A20012) or Alexa Fluor 488 Azide (Thermo Fisher Scientific, #A10266) using Click-It Cell Reaction Buffer kit (Thermo Fisher Scientific, #C10269), according to manufacturer’s protocol

### In vitro Endolysosomal Acidification Assay

U3013 cells were incubated overnight with 30 µg/ml pHrodo Red Dextran (Thermo Fisher Scientific, #P10361). 1 µM FCCP was added to neutralize organelle pH gradient for 15 min and cells were harvested in fractionation buffer (The composition is described in Supplement methods). Cells were mechanically broken down by passing through 23G needle 5 times, then centrifuged at 2000 g for 10 min. The supernatant was equally divided into several aliquots and centrifuged at 20 000 g for 30 min to separate the cytosol (supernatant) and the light organelles (pellet). The pellets were resuspended in 50 µL fractionation buffer. Vacq1 was added together with or without ConA to each well. To stimulate organelle acidification, 2 mM ATP and 2 mM MgCl_2_ were added and incubated for 30 min. The organelle acidification was measured with fluorescence emission of pHrodo Red Dextran. The fluorescence was collected at 590 nm upon 544 nm excitation in a FLUOstar Omega microplate reader

### In Vitro ATPase Assay

U3013 cells were harvested in fractionation buffer and lysed by passing through 23G needle 5 times, then centrifuged at 2000 g for 10 min to discard nucleus. The resulting supernatant were centrifuged at 20 000 g for 30 min. The pellets which include light organelle such as endosome and lysosome were resuspended in fractionation buffer and treated with Vacq1 together with or without ConA. To activate ATPase, 2 mM ATP and 2 mM MgCl_2_ were added and incubated for 30 min. ATPase activity was measured with ADP-Glo Max Assay (Promega, #V7001), according to manufacturer’s protocol.

### Statistical Analysis

Data were statistically evaluated by using GraphPad Prism 9 software. Unpaired two-tailed t-test was consistently used. Statistical significances are depicted as: * *P* ≤ 0.05, ** *P* ≤ 0.01, *** *P* ≤ 0.001, **** *P* ≤ 0.0001. Technical replicates were conducted within experimental replicates. Sample size always indicates experimental replicates.

### Cell Lysis and Immunoblotting

The procedures for cell lysis and immunoblotting are described in the Supplementary Methods.

### KinomeView Profiling

KinomeView profiling procedure is described in the Supplementary Methods.

### Stable Expression of myr-Akt1 and myr-PIK3CA

The procedure for the generation of U3013MG cells stably expressing myr-Akt1 and myr-PIK3CA and plasmids are described in the Supplementary Methods.

### Pull-Down Assay

Vacq1 and CaM pull-down assays are described in the Supplementary Methods.

### Mass Spectrometry

Detailed mass spectrometry analysis is described in the Supplementary Methods.

### Chemical Synthesis of Vacq1 Derivatives

The procedures for chemical synthesis of Vacq1 derivatives are described in the Supplementary Methods.

## Results

### Vacq1-Induced Vacuoles Originate From Plasma Membrane and Targeted to Lysosomes

CADs have previously shown to cause catastrophic vacuolization and this has been considered as a key cellular process by which this class of compounds, including Vacq1, induce glioblastoma cell death. We, therefore, investigated the membrane compartments from which vacuoles are created, and also determined the identity of the accumulating vacuoles. Vacq1 induces strong plasma membrane ruffling, indicating that vacuoles originate from plasma membrane. We labeled the plasma membrane with wheat germ agglutinin (WGA) to see whether Vacq1 (2 µM) resulted in plasma membrane internalization in patient-derived glioblastoma cells (U3013MG).^21^ WGA was readily detected in vacuole membranes of Vacq1-treated cell at 6 hours (Supplementary Figure S1A). WGA was exclusively located in the inner membrane leaflet facing the lumen side of vacuoles, consistent with a formation by an endocytic-like process at the plasma membrane (Supplementary Figure S1A). Monitoring vacuoles with organelles including mitochondria, Golgi, endoplasmic reticulum (ER), and lysosome labeled with GFP-fused organelle-specific proteins to reveal the destiny of newly formed vacuoles. Phase-lucent vacuoles colocalized with the lysosomal marker, but not the other organelles (Supplementary Figure S1B). WGA^+^ lysosomal membranes formed large swollen organelles, i.e. enlarged lysosomal vacuoles (Supplementary Figure S1C). We conclude that Vacq1 induces the formation of plasma membrane-originating vacuoles and the vacuoles eventually fuse with lysosomes.

### Vacq1 Strongly Induces the Formation of Acidic Vesicle Organelles

**Figure 1.**
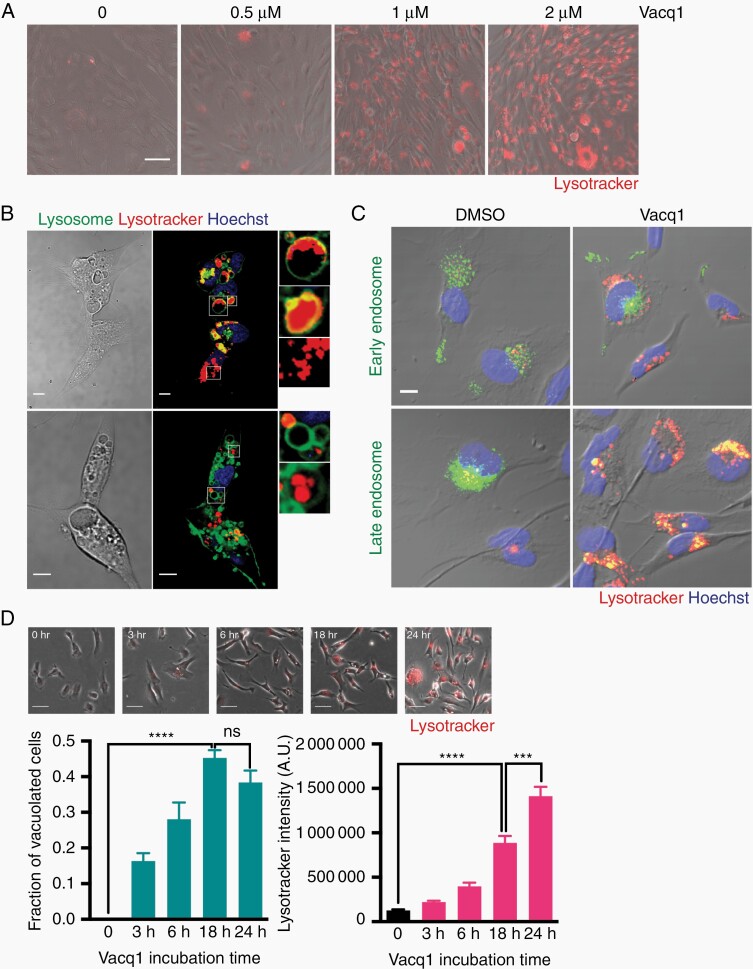
Vacq1 strongly induces the formation of acidic vesicle organelles (AVO) in glioblastoma cells. (A) Analysis of changes in acidic organelles upon Vacq1 treatment at indicated concentrations. Acidic organelles were stained with Lysotracker Red. (B) Cellular distribution of Vacq1-induced lysosomal-enlarged vacuoles and AVO in cells treated with 2 µM Vacq1 for 24 h. Lysosomal vacuoles were labeled with CellLight-Lysosome and AVO were stained with Lysotracker Red. (C) Colocalization of AVOs with late endosome, but not early endosome markers. (D) Temporal dynamics in vacuole and AVO accumulation upon Vacq1 treatment. Top, representative microscopic images of cells at indicated time points. Bottom, quantification of fraction of cells with vacuoles in microscopic field (*n* = 4) and Lysotracker intensity of individual cells (*n* = 109, 127, 265, 175 and 292 for 0, 3 h, 6 h, 18 h and 24 h, respectively, mean ± s.e.m; unpaired two-tailed t-test, *** *P* ≤ 0.001, **** *P* ≤ 0.0001). Scale bars: 50 µm (A), 10 µm (B, C), 100 µm (D).

The finding that Vacq1 induced vacuoles fuse with lysosomes and resulted in enlarged lysosomal vacuoles led us to examine if Vacq1 disrupts lysosomal homeostasis. Since most lysosomal functions are maintained through keeping the lysosomal lumen acidic, we examined if Vacq1 could cause affect lysosome acidification by monitoring pH using Lysotracker Red in glioblastoma cells. Contrary to our expectation, we observed that Vacq1 strongly increased lysotracker fluorescence intensity in a dose-dependent manner (Figure 1A). Detailed analysis revealed that small acidic vesicular organelles (AVO) which have high lysotracker fluorescence intensity were distinct from the enlarged lysosomal vacuoles arising from the plasma membrane (Supplementary Figure S2). Vacq1-induced AVOs mostly existed as separate smaller vesicles, but some AVOs were found inside swollen lysosomes, forming multivesicular bodies (Figure 1B). Monitoring AVOs using endosomal markers revealed that AVOs colocalized with late endosome, but not early endosome (Figure 1C). Interestingly, the kinetics of Vacq1-induced enlarged lysosomal vacuoles and AVOs were different. Phase-lucent enlarged vacuoles were detected from 3 h and saturated within 18 h of exposure, while substantial induction of AVOs occurred between 18 h and 24 h of exposure (2 µM, Figure 1D). We, therefore, conclude that Vacq1 induces the formation of two membranous organelles with different temporal dynamics, lysosomal-enlarged vacuoles and late endosomal AVOs.

### Accumulation of Vacuoles and AVOs Require v-ATPase Activity That Participate in Vacq1-Induced Glioblastoma Cell Death

**Figure 2.**
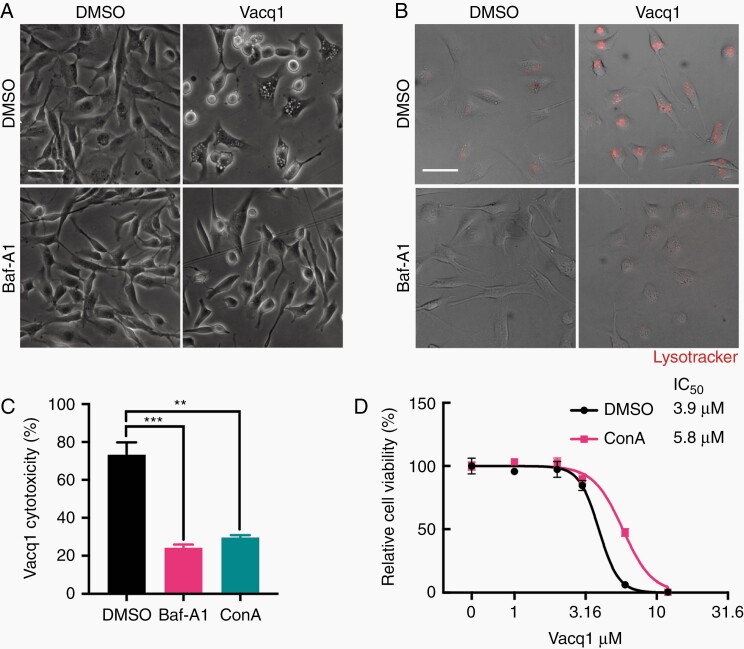
Accumulation of vacuoles and AVOs require v-ATPase activity that participate in Vacq1-induced glioblastoma cell death. (A and B) Effect of vehicle or 10 nM Baf-A1 on Vacq1-induced vacuoles (A) and AVOs (B) accumulation in glioblastoma cells. (C), Cytotoxicity of Vacq1 in the presence of 10 nM Baf-A1 or 10 nM ConA (mean ± s.d., *n* = 3; unpaired two-tailed t-test, ** *P* ≤ 0.01, *** *P* ≤ 0.001). (D) Cell viability of glioblastoma cells treated with increasing doses of Vacq1 in the presence of DMSO or 10 nM ConA (mean ± s.d., *n* = 3). Scale bars: 100 µm (A, B).

The vacuolar-type H^+^-ATPase (v-ATPase) is an ATP-dependent proton pump found in membranes of endosomes and lysosomes where it acidifies the vesicle lumen and is also critical for fusion of the endocytic vacuoles to the lysosomal compartments. The marked and pronounced onset of late endosome acidification resulting in AVO formation by Vacq1 prompted us to examine if v-ATPase is required for vesicle acidification and cytotoxicity in glioblastoma cells. Inhibition of v-ATPase with the specific inhibitor bafilomycin A1 (Baf-A1) abrogated both the formation of Vacq1-induced lysosomal-enlarged vacuoles and AVOs (Figure 2A, B). V-ATPase inhibition with Baf-A1 or concanamycin A (ConA) which blocks v-ATPase–dependent proton translocation^22^ substantially decreased the cytotoxicity of Vacq1 on glioblastoma cells (Figure 2C). Consistently, the half-maximal inhibitory concentration (IC_50_) of Vacq1 on glioblastoma cell viability significantly increased by v-ATPase inhibition with ConA (Figure 2D). Similar results were also obtained using another glioblastoma cell line, U3024MG (Supplementary Figure S3A, B). Thus, we conclude that both Vacq1-induced lysosomal-enlarged vacuoles and AVOs require the v-ATPase and that this activity is required for the cytotoxicity of Vacq1.

Next, we asked if the v-ATPase-sensitive effects of Vacq1 are selective to glioblastoma by examining its effects on primary fibroblast cells. Fibroblast cells exhibited much less vacuolization than glioblastoma cells (Supplementary Figure S3D) and decreased lysotracker fluorescence intensity after exposure to Vacq1, indicating that Vacq1 could not induce AVO formation (Supplementary Figure S3E). Vacq1 was cytotoxic in fibroblast cells, but with higher IC_50_ compared to glioblastoma cells (Figure 2D, Supplementary Figures S3A and S3C). Moreover, v-ATPase inhibition by ConA did not reverse Vacq1-induced cell death in fibroblasts, indicating that Vacq1 toxicity is independent of the v-ATPase (Supplementary Figure S3C). Similar to fibroblasts, ConA did not prevent Vacq1-induced cell death of normal human astrocytes (Supplementary Figure S3F). Taken together, these data indicate that a v-ATPase-sensitive phenotype and cell death by Vacq1 specifically occur in glioblastoma cells.

### Vacq1 Cytotoxicity Through ATP Depletion by Inducing v-ATPase Activity

**Figure 3.**
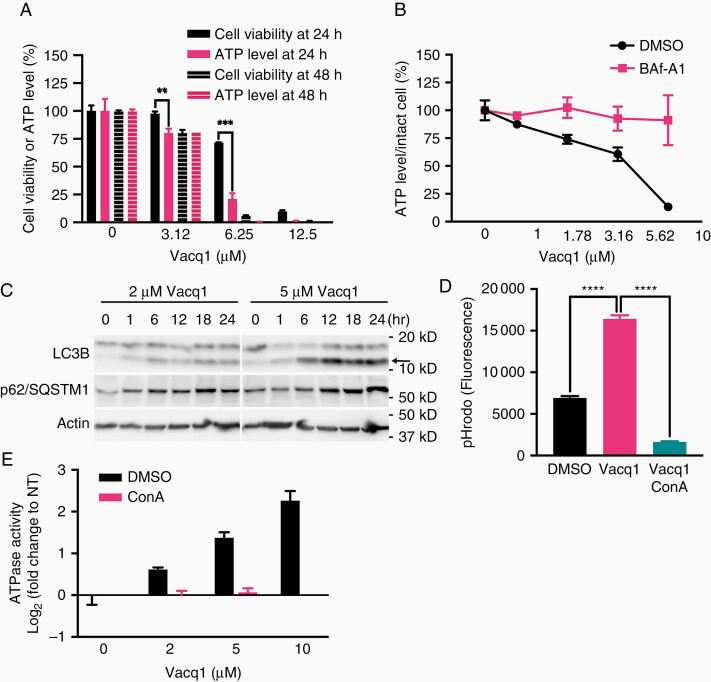
Vacq1 depletes cellular ATP by inducing v-ATPase activity in glioblastoma cells. (A) Glioblastoma cell viability and intracellular ATP levels of cells treated with Vacq1 at 24 h or 48 h (mean ± s.d., *n* = 3; unpaired two-tailed t-test, ** *P* ≤ 0.01, *** *P* ≤ 0.001). (B) Relative ATP level in intact glioblastoma cells exposed to increasing doses of Vacq1 in the presence of DMSO or 10 nM Baf-A1 (mean ± s.d., *n* = 3). (C) Immunoblots of glioblastoma cells exposed to 2 µM or 5 µM Vacq1 for the indicated time periods. Arrow indicates LC3B-II. (D) *In vitro* endolysosomal acidification assay. The pH gradient across the isolated endolysosome membrane was dissipated and reacidification was initiated by adding ATG-Mg in the presence of DMSO, Vacq1 and Vacq1/ConA. The reacidification was monitored as fluorescence of pH sensitive pHrodo which was preloaded before endolysosome isolation (mean ± s.d., *n* = 3; unpaired two-tailed t-test, **** *P* ≤ 0.0001). (E) *In vitro* enzymatic ATPase assay with isolated endolysosomes. ATPase activity in endolysosome membranes were stimulated by adding ATP-Mg in the presence of DMSO, Vacq1 or Vacq1/ConA and activity measured (mean ± s.d., n = 3).

The Vacq1 induced AVOs which become acidified through the activity of v-ATPase indicate that Vacq1 provokes a marked and sudden cellular ATP consumption that could represent one tumor-specific mechanism of Vacq1 toxicity. We therefore first examined if Vacq1 depletes ATP in glioblastoma cells prior to their death. In order to discriminate intact living cells from dead cells, we measured total cellular ATP while at the same time monitoring cell viability by parallel measurements of protease activity released from dead cells. Reduction of total ATP level and cell viability were indifferent at 48 h of Vacq1 exposure at several concentrations, indicating that total ATP levels are a direct reflection of cell viability at this time-point (Figure 3A). However, at 24 h of exposure of 3.12 µM and 6.25 µM Vacq1, ATP was markedly depleted while viability was relatively unchanged (Figure 3A). This result indicates that ATP depletion precedes cell death upon Vacq1 treatment. Consistently, Vacq1 significantly reduced the relative ATP level of intact cells in a dose-dependent manner, and Baf-A1 completely reversed the depletion (Figure 3B). ATP depletion activates autophagy to replenish energy in cells. Consistent with Vacq1-induced ATP depletion, representative autophagy markers, LC3B-II and p62 accumulated in the presence of Vacq1, indicating an increase in the autophagic process (Figure 3C). Next, we asked whether Vacq1 could directly induce v-ATPase activation and consequent acidification. Adopting a cell-free assay system for quantification of ATPase activity using an *in vitro* endolysosomal acidification assay^23^ on the isolated endolysosomal fraction revealed that Vacq1 strongly increased organelle acidification, and ConA blocked the increase (Figure 3D). This indicates that Vacq1 directly act on endolysosomal organelles to cause acidification mediated by the v-ATPase. In agreement with this, Vacq1 enhanced ATPase activity in a dose-dependent manner and ConA abolished the increase in an ATPase assay with the endolysosomal organelle fraction, indicating that v-ATPases on organelle membranes are activated in the presence of Vacq1 (Figure 3E).

### A Critical Role of Protonation and Trapping of Vacq1 Within AVOs for Glioblastoma Toxicity

Vacq1 is an amphiphilic amine. It has a secondary amine that can be protonated at acidic pH. Thus, Vacq1 could be trapped and accumulate in the acidic compartment once it is protonated in the lumen of the compartment. This property opens for that Vacq1-induced AVOs in glioblastoma cells represent a reservoir of high quantities of accumulated Vacq1. In order to examine the accumulation of the compound in living cells, we synthesized a functional analog (which we named Vacq1-Click) that allowed the use of click chemistry to visualize Vacq1 molecules *in situ* in the cells (Supplementary Figure S4A). Vacq1-Click retained the functional properties of Vacq1 as it induced enlarged vacuole formation and AVO induction which could be blocked by v-ATPase inhibition using Baf-A1 (Supplementary Figure S4B). Vacq1-Click also had comparable cytotoxicity to Vacq1 which was attenuated by Baf-A1 (Supplementary Figure S4C). Combined these results indicate that Vacq1-Click can be used to examine the cellular localization of the compound in cells. We found a dose-dependent accumulation of Vacq1-Click in puncta in glioblastoma cells (Supplementary Figure S4D) and blocking v-ATPase by Baf-A1 completely prevented cellular accumulation of Vacq1-Click (Supplementary Figure S5A), showing that organelle acidification by v-ATPase is required for Vacq1-Click accumulation. Furthermore, Vacq1-Click accumulation in the punctate form occurred at late time (24 h) after Vacq1 administration with much less accumulation at 4 h (Supplementary Figure S4E). Because AVOs are more acidic and form later than lysosomal-enlarged vacuoles (Figure 1D), this result is consistent with that Vacq1 accumulates in AVOs. We confirmed that Vacq1-Click localizes to the AVOs, as it colocalized both with lysotracker and the late endosome, but not early endosome (Supplementary Figure S5B, C). We, therefore, conclude that Vacq1 activates v-ATPase activity which induces the formation of AVOs and thereafter accumulates in these vesicles.

This finding opened for that the accumulation of Vacq1 in the induced AVOs is a necessary feature of the compound’s action. Because protonation is required for trapping of amphiphilic amines in acidic environments, we compared the cytotoxicity of Vacq1 to that of several derivatives which have different pKa (Supplementary Figure S6A). Among these derivatives, CBK277826 cannot be protonated in acidic organelle (pKa of 4.32), while all other derivatives together with Vacq1 can be readily protonated. All protonatable derivatives showed comparable cytotoxicity to Vacq1, but CBK277826 was much less cytotoxic (Supplementary Figure S6B). Even at the highest concentration (50 µM) of CBK277826 used, ~40% glioblastoma cells remained alive (Supplementary Figure S6B). This indicates that protonation of Vacq1 and subsequent accumulation in AVOs is critical for Vacq1-induced glioblastoma cell death. If the accumulation of Vacq1 is crucial for Vacq1-induced cell death, the incorporated molecular number per cell should be a decisive factor. Thus, the cytoxicity should be directly related to the absolute quantity rather than concentration of Vacq1. Changing the molecular quantity of Vacq1 while keeping the same concentration by adding different volumes of Vacq1-containing medium showed that the number of dead cells (round shape) and cells containing vacuoles was directly proportional to the quantity of Vacq1 molecules, but not to Vacq1 concentrations (Supplementary Figure S5D). Furthermore, maintaining concentration but increasing volume resulted in substantial shift of IC_50_ in a dose-response (Supplementary Figure S5E). We then tested if the quantity-dependent enhancement in the cytotoxicity of Vacq1 could be due to its accumulation in AVOs. We found that the enhanced cytotoxicity accompanied by increasing the molecular quantity was largely reversed by preventing Vacq1 accumulation using ConA inhibition of the v-ATPase (Supplementary Figure S5F). Taken together, these data suggest that the trapping of Vacq1 in AVOs crucially contributes to cell death.

### Profiling Signaling Pathways Reveal That Vacq1’s Suppression of Akt is Dispensable for Vacq1-Induced Endolysosomal Disruption and Glioblastoma Cell Death

The previous results explain the mechanism by which Vacq1 contributes to cell death through activation of the v-ATPase in AVO. In order to identify the mechanism of action of Vacq1 in the other compartment, the lysosomal-enlarged vacuoles, we performed antibody-based profiling of post-translational modification to capture signaling pathways that are affected upon Vacq1 treatment (Supplementary Figure S7). We found that Vacq1 suppresses phospho-Akt (p-Akt) as well as phosphorylation of Akt substrate motif showing that Vacq1 strongly inhibits the Akt pathway (Supplementary Figures S7 and S8A). We examined if Akt inhibition could be a downstream event of endolysosomal disruption by Vacq1. Since Vacq1-induced endolysosomal alteration is completely abolished by v-ATPase inhibitor, we examined p-Akt status in the presence of Baf-A1. We found that Vacq1 could suppress p-Akt regardless of the presence of Baf-A1 (Supplementary Figure S8B). Next, we tested if Akt suppression is required for endolysosomal disruption. The results showed that glioblastoma cells expressing constitutively active Akt (Supplementary Figure S8D) increased AVOs upon Vacq1 treatment, similar to control cells (Supplementary Figure S8C). These results indicate that Akt inhibition and endolysosomal disruption are mutually independent responses to Vacq1 treatment. Because of this, we asked whether Akt inhibition is dispensable or if it contributes to Vacq1-induced cell death using a constitutively active form of Akt or its upstream PI3K. The results showed that the constitutively active Akt constructs were unable to rescue from Vacq1-induced glioblastoma cell death (Supplementary Figure S8D, E), indicating that even though Vacq1 attenuates Akt signaling, this effect does not contribute to Vacq1-induced cell death.

### Chemical Proteomics Reveal a Direct Interaction of Vacq1 With Calmodulin

Because we could not identify any signaling pathway which could explain the mechanisms of action of Vacq1 on lysosomal-enlarged vacuoles, we instead used a chemical proteomics approach to identify target proteins of Vacq1. Using a sepharose-bead immobilized derivative of Vacq1, we enriched Vacq1-interacting proteins from two different glioblastoma cell lysates (U3013MG or U3024MG) (Supplementary Figure S9A). To find specific Vacq1-interacting protein, we first immobilized Vacq1-captured proteins and then washed with excess amounts (10 mM) of free Vacq1 followed by high-resolution mass spectrometry analysis. We found 469 proteins that were captured on the immobilized Vacq1 (Supplementary Table 1) and 124 of these were specifically competed out (> 50%) with free Vacq1 (Supplementary Figure S9B). Among these, one of the top-ranked proteins was calmodulin (CaM) (Supplementary Figure S9B). Immunoblot analysis confirmed the direct interaction between Vacq1 and CaM in two different glioblastoma cell lines (Supplementary Figure S9C). CaM transmits Ca^2+^ signal through Ca^2+^-dependent interaction with signaling proteins such as CaMKII and mTOR.^24^ We asked if Vacq1 could directly perturb the interaction between CaM and its functional binding proteins by performing pulldown of CaM-sepharose after incubation with cell lysate and examined the level of CaM-binding proteins, CaMKII, and mTOR in the precipitates. We found that CaM specifically interacts with mTOR and CaMKII in a Ca^2+^-dependent manner and Vacq1, as well as the structural analogs (CBK277826 and CBK277852) but not chloroquine, interfered with this interaction in a dose-dependent manner, with similar potency to W7, which is a previously known CaM antagonist (Supplementary Figure S9D, E). These results indicate that Vacq1 interacts with and has antagonistic effects on CaM.

### Vacq1 Causes a Failure of Endolysosomal Reformation by Fission Through CaM Inhibition

**Figure 4.**
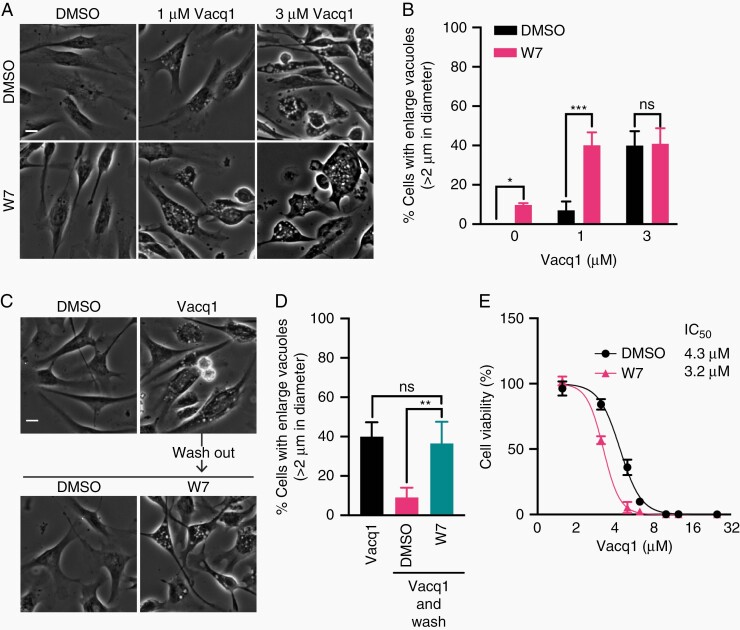
Vacq1 causes a failure of endolysosomal reformation by fission through CaM inhibition. (A) Micrographs of glioblastoma cells treated with Vacq1 at indicated concentrations together with DMSO or CaM inhibitor W7 (10 µM). (B) Quantification of the percentage of glioblastoma cells with enlarged vacuoles shown in A (mean ± s.d., *n* = 4; unpaired two-tailed t-test, * *P* ≤ 0.05, *** *P* ≤ 0.001). (C) Glioblastoma cells were treated with Vacq1 for 5 h and then washed and replaced with fresh medium with or without W7 (10 µM). (D) Quantification of the percentage of glioblastoma cells with enlarged vacuoles shown in B (mean ± s.d., *n* = 4; unpaired two-tailed t-test, ** *P* ≤ 0.01). (E) Viability of glioblastoma cells exposed to increasing concentrations of Vacq1 in the presence of DMSO or 10 µM W7 (mean ± s.d., *n* = 3). Scale bars: 20 µm.

**Figure 5.**
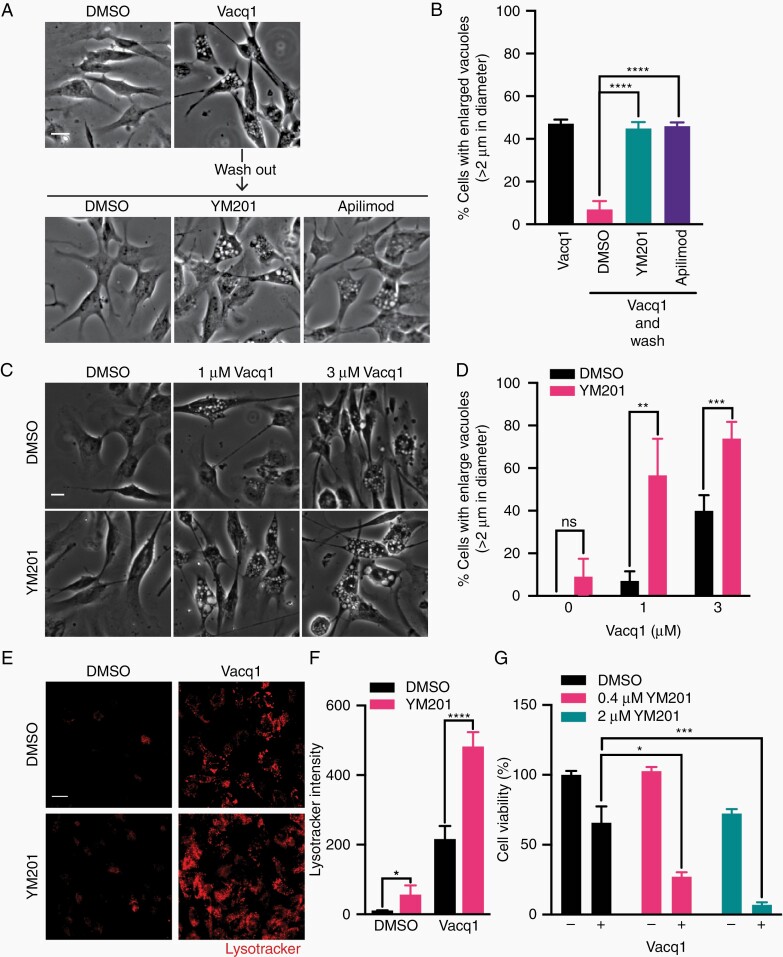
Combined treatment of PIKfyve inhibitor and Vacq1 leads to a disruption of lysosomal reformation and prevents clearance of AVOs. (A) Glioblastoma cells were treated with Vacq1 for 5 h and then washed and replaced with fresh medium with or without inhibitors of PIKfyve (YM201, 200 nM; Apilimod, 10 nM) which is required for endolysosomal fission. (B) Quantification of the percentage of glioblastoma cells with enlarged vacuoles shown in A (mean ± s.d., *n* = 4; unpaired two-tailed t-test, *** *P* ≤ 0.0001). (C) Micrographs of glioblastoma cells treated with Vacq1 together with DMSO or YM201 (200 nM). (D) Quantification of the percentage of glioblastoma cells with enlarged vacuoles shown in C (mean ± s.d., *n* = 4; unpaired two-tailed t-test, ** *P* ≤ 0.01, *** *P* ≤ 0.001). (E) Vacq1-induced AVO accumulation detected by lysotracker in the presence of DMSO or YM201 (200 nM). (F) Quantification of lysotracker fluorescence intensity of E (mean ± s.d., *n* = 4; unpaired two-tailed t-test, * *P* ≤ 0.05, **** *P* ≤ 0.0001). (G) Cytotoxicity of Vacq1 in the presence of YM201 at indicated concentrations (mean ± s.d., *n* = 3; unpaired two-tailed t-test, * *P* ≤ 0.05, *** *P* ≤ 0.001). Scale bars: 20 µm.

CaM is intimately involved in the lysosome membrane fission process and its blockage induces enlarged lysosomal vacuoles, similar to those induced by Vacq1.^25^ Therefore, we hypothesized that Vacq1 could prevent lysosome fission by the inhibition of CaM, resulting in the accumulation of large lysosomal vacuoles. To test this, we first examined the effect of W7 on vacuole formation by Vacq1 treatment. W7 potentiated the formation of large vacuoles at a submaximal dose of Vacq1 (1 µM) (Figure 4A, B). However, W7 alone did not substantially increase vacuolated cells implying that CaM inhibition is not by itself sufficient for inducing vacuole formation (Figure 4A, B). When 3 µM Vacq1 was used, the co-treatment with W7 did not lead to any further increase, indicating that Vacq1 fully inhibits CaM at 3 µM (Figure 4A, B). These findings indicate that Vacq1 not only causes the initiation of vacuole formation by the induction of macropinocytosis but also blocks the clearance of vacuoles by inhibiting lysosome fission through the regulation of CaM, resulting in the accumulation of lysosomal vacuoles. To test this possibility, we performed a pulse-chase experiment in which lysosomal-enlarged vacuoles first were induced by Vacq1, cells were thereafter washed free of Vacq1 and clearance of lysosomal vacuoles was measured with and without inhibition of CaM by W7. The clearance of vacuoles was rapid, with a reduction of vacuolated cells from ~40% to ~10% after 1 h of Vacq1 removal (Figure 4C, D). However, W7 treatment completely prevented the clearance of vacuoles (Figure 4C, D). This result suggests that CaM inhibition by Vacq1 prevents lysosomal vacuole fission. If W7 and Vacq1 act by a similar mechanism on lysosomal-enlarged vacuoles and this contributes to glioblastoma cytotoxicity, they are expected to have additive effects on cell cytotoxicity at submaximal inhibition. W7 led to vacuolization at lower doses of Vacq1 and reduced the IC50 of Vacq1 in a dose-response assay on cell viability (Figure 4E). Endolysosomal fission requires the enzyme PIKfyve, a kinase that synthesizes the phosphoinositide phosphatidylinositol 3,5-bisphosphate (PI(3,5)P2) in endosome membranes which is necessary for lysosome fusion and fission.^26,27^ Consistently, PIKfyve is also critical for lysosomal membrane trafficking following macropinocytosis^28,29^ and loss of function of PIKfyve causes lysosomal coalescence observed as fewer but enlarged lysosomal vacuole accumulation with a failure of lysosomal reformation by fission.^27,29^ We, therefore, examined if PIKfyve inhibition prevents the recovery of lysosomal membrane from Vacq1-induced lysosomal vacuoles and found that YM201636 (YM201) and apilimod, which are selective PIKfyve kinase inhibitors,^30,31^ prevented the clearance of vacuoles after removal of Vacq1 (Figure 5A, B). Furthermore, YM201 profoundly potentiated the effect of 1 µM Vacq1 on vacuolization (Figure 5C, D) and in contrast to the CaM inhibitor W7, also increased vacuolization at 3 µM of Vacq1 (Figure 5C, D). This shows that CaM and PIKfyve act in the same Vacq1-induced membrane trafficking compartment through a mechanism of preventing lysosomal reformation, however, PIKfyve inhibition exerts more profound perturbation of lysosome reformation than CaM inhibition by Vacq1 or W7.

### Disruption of Lysosomal Reformation Prevents Clearance of AVOs

Our previous results show that glioblastoma-specific cytotoxicity of Vacq1 involves an activation of the v-ATPase in AVOs, resulting in ATP depletion and metabolic catastrophe. However, it remained unclear how the Vacq1-induced inhibition of CaM with the resulting enlarged lysosomal vacuoles contribute to its cytotoxicity. AVOs are formed through acidification of endosomes which are cleared through lysosomal fusion forming endolysosomes. Thus, depletion of lysosomes through Vacq1-induced CaM inhibition could prevent the clearance of endosomes and thereby contribute to the accumulation of endosomes in which Vacq1 is captured and forms AVOs by the activation of the v-ATPase. To examine this possibility, we took advantage of the robust formation of enlarged lysosomal vacuoles by PIKfyve inhibition. Consistent with the physiological importance of lysosomes for endosome recycling, YM201 inhibition of PIKfyve resulted in a small but significant increase of AVOs (Figure 5E, F). However, when combined with Vacq1, it led to a marked and synergistic elevation of AVOs, similar to the effect of the combination on lysosomal vacuole formation (Figure 5E, F). This result implies that enlarged lysosomal vacuole accumulation positively regulates AVO formation through a depletion of lysosomes. Consistent with these results, YM201 synergistically increased the cytotoxic potency of Vacq1 in a cell viability assay (Figure 5G).

## Discussion

Vacq1 is known to perturb endocytic-like membrane trafficking, induce macropinocytosis and lead to catastrophic vacuolization and lysis of glioblastoma cells.^17,18^ Vacq1 has potential as a new drug for glioblastoma, but unfortunately is also associated with general toxicity that limit clinical utility. The objective of this study was to identify the mechanism of action of Vacq1, and by this open for rational strategies to develop compounds eliminating the nonselective toxicity while retaining glioblastoma-specific cytotoxicity.

**Figure 6.**
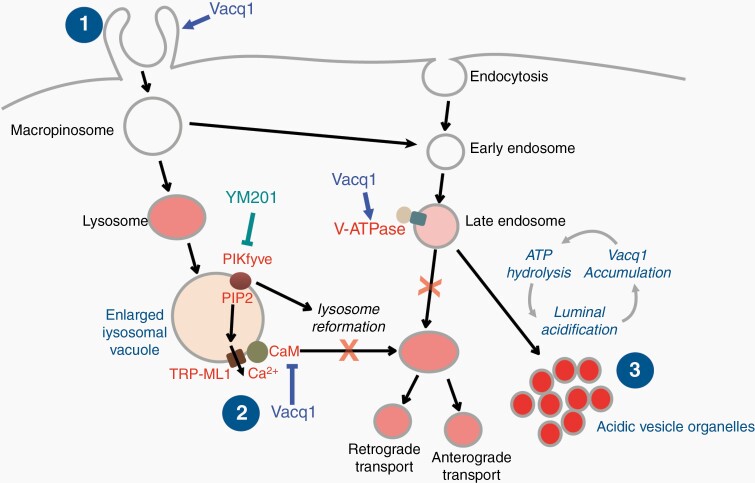
Schematic illustration of proposed mechanism of action of Vacq1-induced endolysosomal disruption and glioblastoma cell death. Vacq1 initiates plasma membrane blebs and ruffles and this activity leads to macropinocytic endocytosis. The newly formed vesicles have two distinct destinations. One pool is fused with lysosomes forming enlarged vacuoles. Vacq1 directly interacts and interferes with CaM. The inhibition of CaM prevents lysosomal reformation from the vacuoles, and consequently enlarged vacuoles accumulate. Within endosomes, Vacq1 activates v-ATPase which causes an abnormal acidification and formation of AVOs. Protonation and trapping of Vacq1 within the lumen of the acidic organelles contributes to glioblastoma cytotoxicity.

The glioblastoma tumor inter- and intraheterogeneity of oncogenic mutations and the variety of transcriptional properties and epigenetic modifications have posed enormous difficulties to identify single specific targets that effectively limit tumor propagation and recurrence, and thus, the classic treatment with surgery, radiotherapy, and/or chemotherapy remain the standard therapy.^32–34^ Instead of narrowly treating specific mutations and subtypes, strategies looking for therapies that address the shared biology of gliomas might be more successful. Thus, targeting the cancer phenotype that unify this disease rather than those that appear to subdivide it.^35^ This idea has spurred a revival for phenotypic screens with the intention of identifying acquired dependencies on cellular processes/pathways that can be exploited for development of new strategies for the treatment of glioblastoma. Intriguingly, a number of small molecules with the shared features of being cationic amphiphilic compounds are highly cytotoxic to glioblastoma. Cationic amphiphilic compounds are a wide group of chemicals characterized by common structural features but with multiple different mechanisms of action. For example, chloroquine and chloroquine-like drugs have attracted attention for their cytotoxicity and synergizing properties with radiation and temozolomide to promote demise of glioblastoma.^36,37^ Unlike these, which de-acidify endolysosomes, another set of compounds share the cellular phenotype of inducing macropinocytosis and deregulation of endolysosomal trafficking pathway, leading to the formation of massive numbers of empty, variable-sized, intracellular vacuoles which eventually leads to nonapoptotic cell death.^12,17,38,39^ The protein targets and detailed mechanisms of these compounds are not known. In this study, we have used variety of technological strategies and identified an inhibitory interaction of Vacq1 with CaM and an agonistic activation of v-ATPase in the endolysosomal trafficking pathway as the responsible molecular mechanisms and targets for Vacq1-induced lysosomal vacuolization and AVO formation, respectively. Our results are consistent with that the Vacq1-induced forced formation of AVOs with the accompanying ATP depletion leads to a metabolic catastrophe and that this is the critical effector of glioblastoma cell death. In contrast, inhibition of CaM by itself has little effect on glioblastoma survival unless in the context of AVO formation. Thus, we find that in cells with forced formation of AVOs, the inhibition of lysosome reformation by CaM inhibition prevents clearance of late endosomes and shows that this contributes to the cytotoxicity of Vacq1. Therefore, a simultaneous interference by Vacq1 at multiple sites of the endolysosome pathway (Figure 6) appears to be a key feature underlying the beneficial cytotoxic effect, and which can provide selectivity to glioblastoma cells. We discuss these conclusions in greater detail below.

Plasma membrane ruffling and initiation of macropinocytosis is likely an initiating event for Vacq1 cytotoxicity. Although difficult to address experimentally due to the lack of methods to specifically target v-ATPase on the plasma membrane, a recent study reported that the plasma membrane v-ATPase is indeed required for macropinocytosis.^40^ Thus, it seems possible that the activation of v-ATPase on plasma membrane also could be a critical starting event by Vacq1. However, our results reveal a more complex cytotoxic mechanism of action of Vacq1, involving at least two intracellular targets. Our data suggest that the accumulation of enlarged lysosomal vacuole formation is the result of blockage of the lysosome fission process and we present CaM as a responsible molecular target for that. We used mass spectrometry in an unbiased whole proteomic analysis to identify Vacq1 interactors. The prototypical Ca^2+^ sensory calmodulin was identified, and Vacq1 was independently confirmed to interact with calmodulin and to act as an inhibitor, comparable to the known calmodulin inhibitor W7. CaM is activated by lysosomal Ca^2+^ release and triggers lysosome fission.^25^ In agreement with the role of CaM in the lysosome fission, we found that W7 has additive effect on enlarged vacuole formation only in subthreshold concentration of Vacq1. Furthermore, W7 prevented vacuole clearance after Vacq1 removal. These results suggest that Vacq1 prevents lysosome fission through CaM inhibition which leads to the accumulation of enlarged lysosomal vacuoles. Even though our data suggest that CaM inhibition is necessary for Vacq1-induced enlarged vacuole formation, blocking CaM is not sufficient by itself, since W7 alone did not lead to vacuole formation. Consistently, PIKfyve inhibitor (YM201) which has a profound effect on lysosome recovery did not cause the formation of enlarged vacuoles, but similar to W7 prevented clearance of Vacq1-induced vacuoles. Thus, consistent with previous results showing that CADs require macropinocytic vacuolization,^12,13,17^ we found Vacq1-induced a plasma membrane flow and formation of macropinocytic enlarged vacuoles. In addition to this activity, our data support that CaM inhibition by Vacq1 in enlarged lysosomal vacuoles contributes to its cytotoxic properties. However, the enlarged lysosomal vacuoles seem not directly conferring glioblastoma cytotoxicity, since they occur within a few hours of Vacq1 exposure, when most cells are still intact and alive. Nevertheless, the blockage of lysosome reformation from vacuoles could be a cause of the accumulation of late endosomal AVOs, since it results in the depletion of functional lysosomes. Functional lysosomes are required for the disposal of late endosome. This would explain why enlarged lysosomal vacuoles and late endosomal AVOs appear sequentially. In agreement with this conclusion, further blockage of lysosome reformation with YM201 caused a synergistic augmentation of AVOs.

In this study, we suggest that Vacq1 act on the v-ATPase in endosomal membranes and that this is the mechanism driving the formation of AVOs. Our experiments using the endolysosome acidification assay showed that Vacq1 activates the v-ATPase and that this leads to the acidification. The accumulation of Vacq1 through protonation in late endosomes appears to be a mechanism that is required for the acidification, since only structural analogs that can be protonated in the acidic organelle were found to cause the formation of AVOs and to confer glioblastoma cytotoxicity (Supplementary Figure S6C). These data suggest that Vacq1 causes a vicious cycle, whereby protonated Vacq1-mediated v-ATPase activation promotes organelle acidification which leads to Vacq1 protonation. This loop results in constitutive v-ATPase activation and consequent ATP consumption. Consistently, we found that Vacq1 depleted cellular ATP prior to cell death. The v-ATPase specific inhibitors, Baf-A1 or ConA completely blocked ATP depletion and reversed Vacq1 cytotoxicity in glioblastoma cells. Thus, we conclude that metabolic catastrophe from cellular ATP depletion is one of the main causes of glioblastoma cell death by Vacq1.

The exact molecular mechanisms for v-ATPase activation by Vacq1 remains unknown. However, protonation and accumulation CADs can lead to up to 1000-fold increased concentration.^41^ Consistently, we found very high concentrations of Vacq1 in the v-ATPase containing endosomal compartments using functionalized Vacq1, which allowed for the direct imaging of the compound within the cells. The v-ATPase contains a soluble cytosolic V_1_ sector that hydrolyzes ATP and a membrane integral V_0_ sector responsible for proton transport. V-ATPase activity is regulated by the reversible dissociation of the V_1_ from the V_0_ sector. Furthermore, association and activity are tightly regulated in response to various stimuli such as nutrients, hormones and growth factors.^42,43^ In terms of molecular mechanisms of v-ATPase activation, phosphatidylinositol phosphate (PIP) lipids content in the organelle membrane has emerged as a central feature. In yeast, V_1_ sector assembly and stability is regulated by phosphatidylinositol 3,5-bisphosphate (PI(3,5)P_2_) through interactions with the V_0_ sector.^44^ Interestingly, similar to our results on Vacq1, PI(3,5)P_2_ increases both the v-ATPase activity and proton pumping activity in isolated vacuolar vesicles.^45^ The similarities in functional effects by Vacq1 and PI(3,5)P_2_ on the v-ATPase in isolated vacuoles, their concentration in the endosomal membrane compartments, and their shared amphiphilic nature could indicate that they also share some molecular principles for v-ATPase activation.

Our dose-response studies show that fibroblasts require higher concentration of Vacq1 for cell death than glioblastoma cells. However, the endolysosomal disruption by Vacq1 is selective to glioblastoma cells as Vacq1 did not cause accumulation of enlarged vacuoles or AVOs in fibroblast, nor could v-ATPase blockage in fibroblasts and normal human astrocytes rescue from Vacq1-induced cell death. Therefore, Vacq1-induced cell death in fibroblasts and astrocytes is caused by an unrelated general cytotoxicity.

This study identifies a dual glioblastoma-specific cytotoxicity of Vacq1 which includes both an activation of v-ATPases in the endosomal compartment as well as an interaction of Vacq1 with CaM which exacerbates the toxicity by interfering with clearance of the AVOs through lysosome depletion. The identification of the mode of action of Vacq1 opens for the development of more specific candidate drugs with robust in vivo efficacy.

## Supplementary Material

vdab152_suppl_Supplementary_Figures

vdab152_suppl_Supplementary_Table_S1

## Notes

**Authorship Statement**. D.K. and P.E. conceived the study, designed experiments and analyzed results. D.K. carried out the experiments; L.G.J.H and M.H. synthesized Vacq1 and its derivatives. D.K. and P.E. wrote the manuscript.
