# Supplementary material for: Glioblastoma cytotoxicity conferred through dual disruption of endolysosomal homeostasis by Vacquinol-1
Source: Neurooncol Adv. 2021 Oct 15;3(1):vdab152. doi: 10.1093/noajnl/vdab152 (PMC8577523; doi:10.1093/noajnl/vdab152)
Supplement: vdab152_suppl_Supplementary_Figures [file vdab152_suppl_supplementary_figures.pdf]

SUPPLEMENTARY INFORMATION

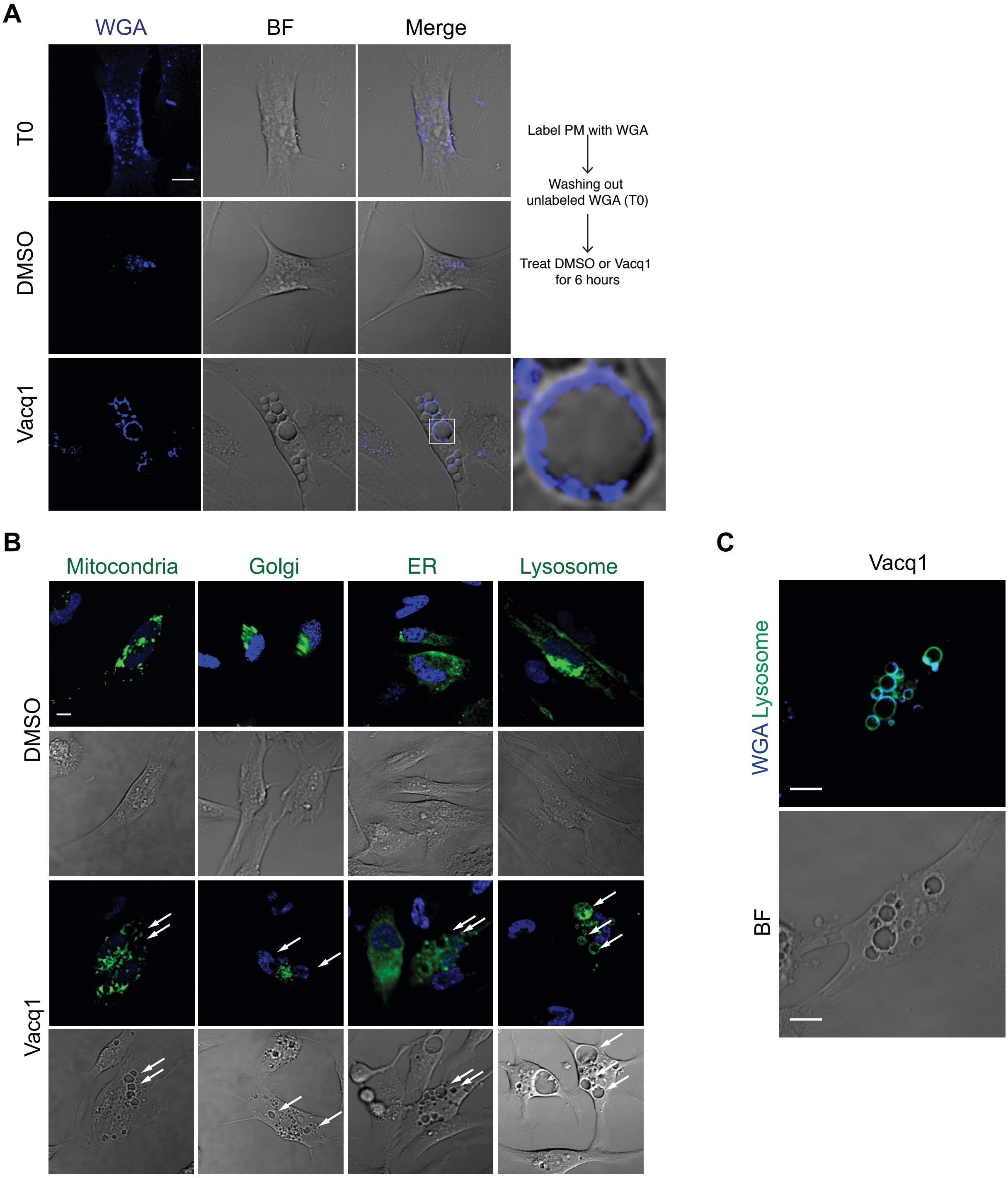

Figure S1 Vacq1-induced enlarged vacuoles originate from plasma membrane that targets to lysosomes. (A) Pulse-chase monitoring of plasma membrane in U3013 glioblastoma cells. Plasma membrane labeled with WGA and chased for 6 h after vehicle (DMSO) or 2 µM Vacq1. (B) Colocalization of organelle markers and Vacq1-induced enlarged vacuoles. Arrows indicate vacuoles. (C) Colocalization of internalized plasma membrane labeled with WGA and lysosome membrane. Scale bars: 10 µm (A-C).

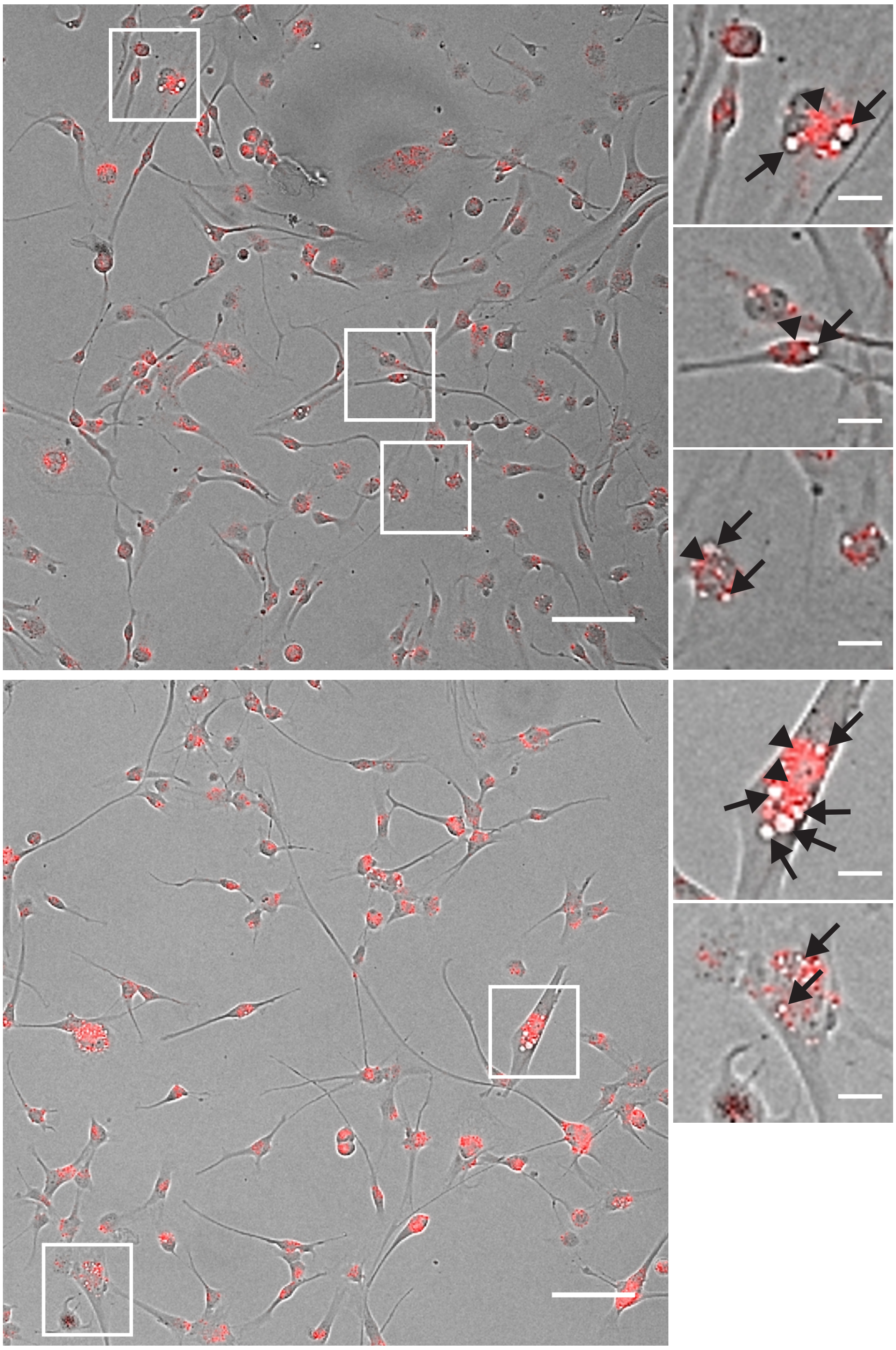

Figure S2 Vacq1 induces two distinct abnormal organelles, enlarged vacuoles and acidic vesicular organelles. Representative micrographs of glioblastoma cells treated with 2 μM Vacq1 for 24 h. AVOs were stained with lysotracker. Inset, arrowheads indicate AVOs and arrows enlarged vacuoles. Scale bar: 50 μm, 10 μm (inset).

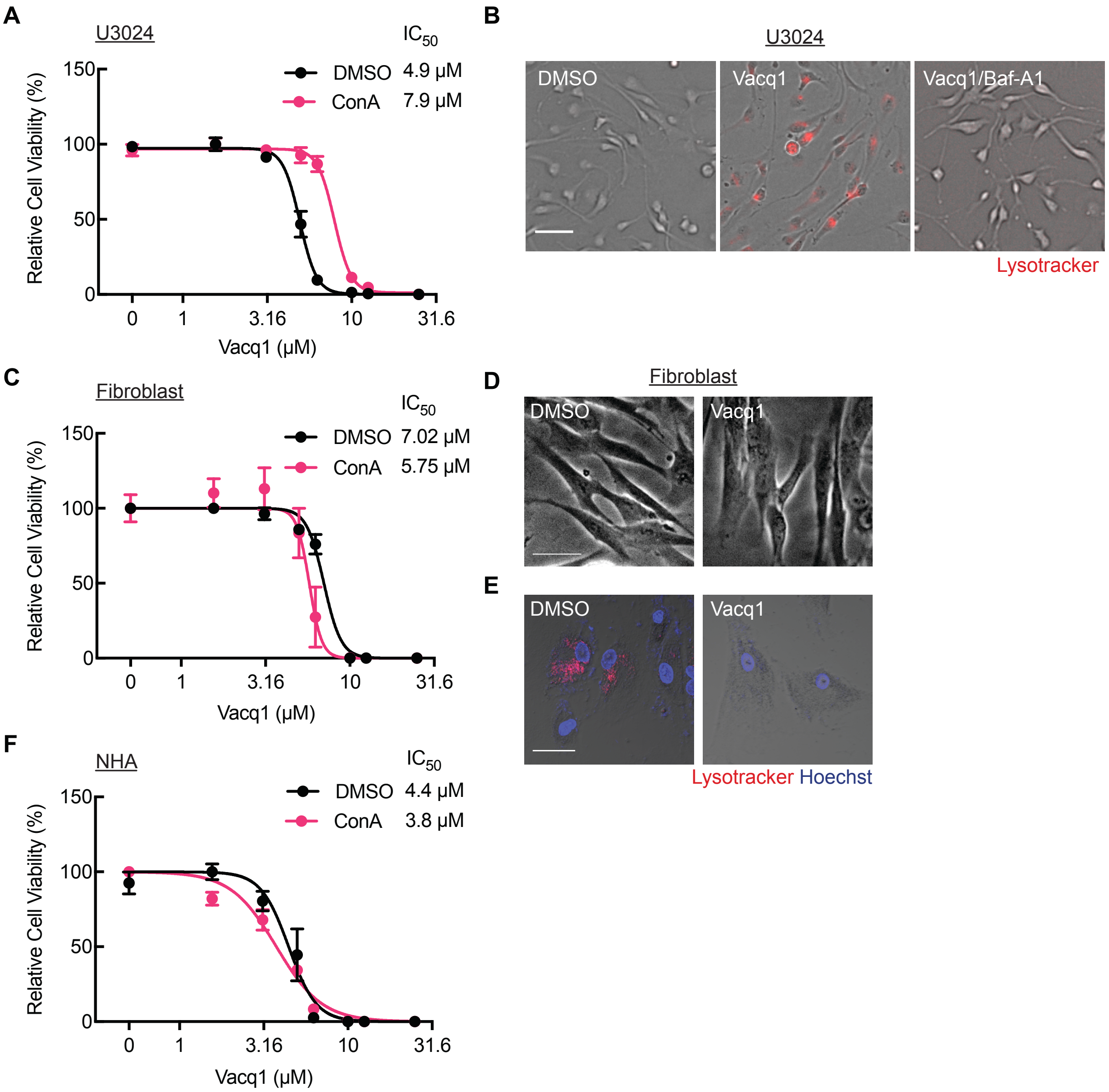

Figure S3 Vacq1 specifically causes v-ATPase-dependent cytotoxicity in glioblastoma cells not in fibroblast and astrocyte. (A, C and F) Cell viability of glioblastoma cells, U3024MG (A), normal human fibroblast (C) or normal human astrocyte (NHA) (F) treated with increasing doses of Vacq1 in the presence of DMSO or 10 nM ConA (mean ± s.d., n = 3). (B) Effect of Vacq1 in U3024MG on AVOs accumulation. (D and E) Effect in fibroblasts of Vacq1 on vacuole formation (D) and AVOs accumulation (E). Scale bars: 100 μm (B), 50 μm (E, F).

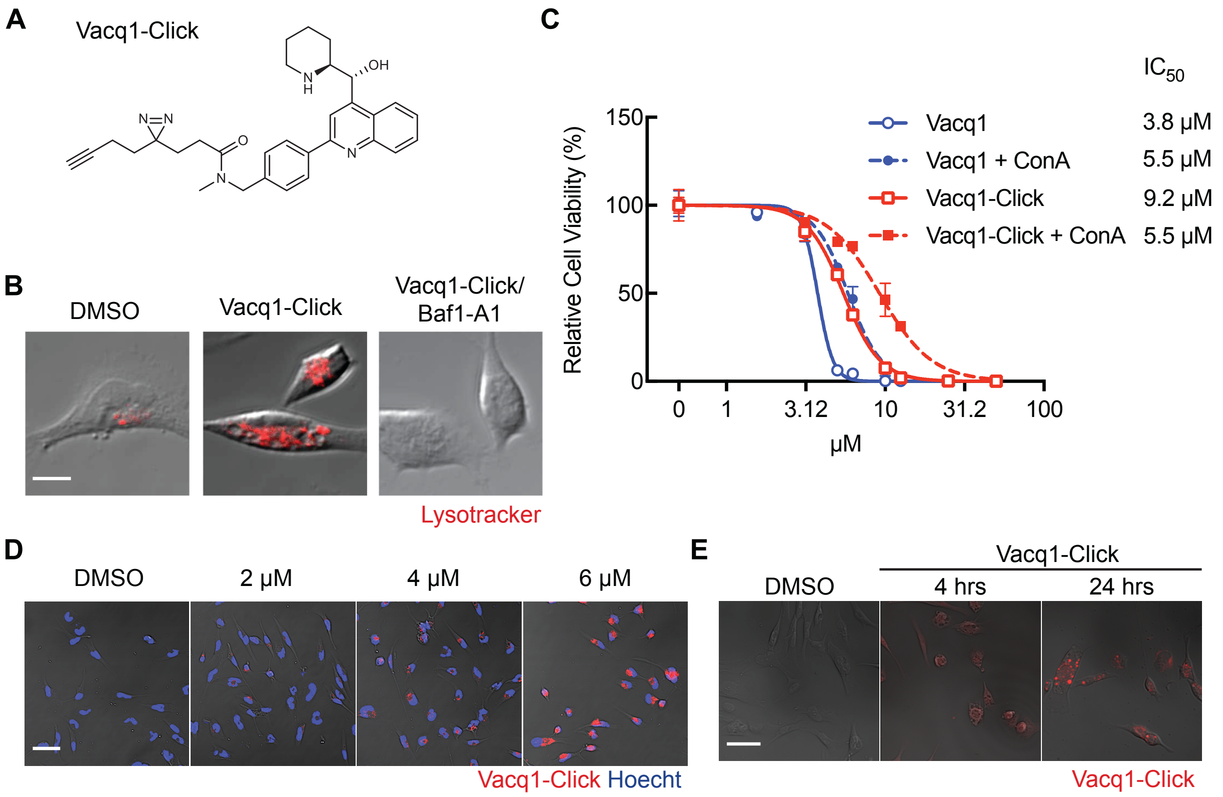

Figure S4 Vacq1-Click retains the functional properties of Vacq1. (A) Chemical structure of Vacq1-Click. (B) Induction of AVOs by Vacq1-Click (5 μM) in the presence or absence of Baf-A1 (10 nM). (C) Cell viability of glioblastoma cells exposed in increasing doses of Vacq1 or Vacq1-Click in the presence of DMSO or 10 nM Baf-A1 (mean ± s.d., n = 3). (D and E) Dose dependency (D) and temporal dynamics (E) of intracellular accumulation of Vacq1-Click. Scale bars: 10 μm (B), 50 μm (D, E).

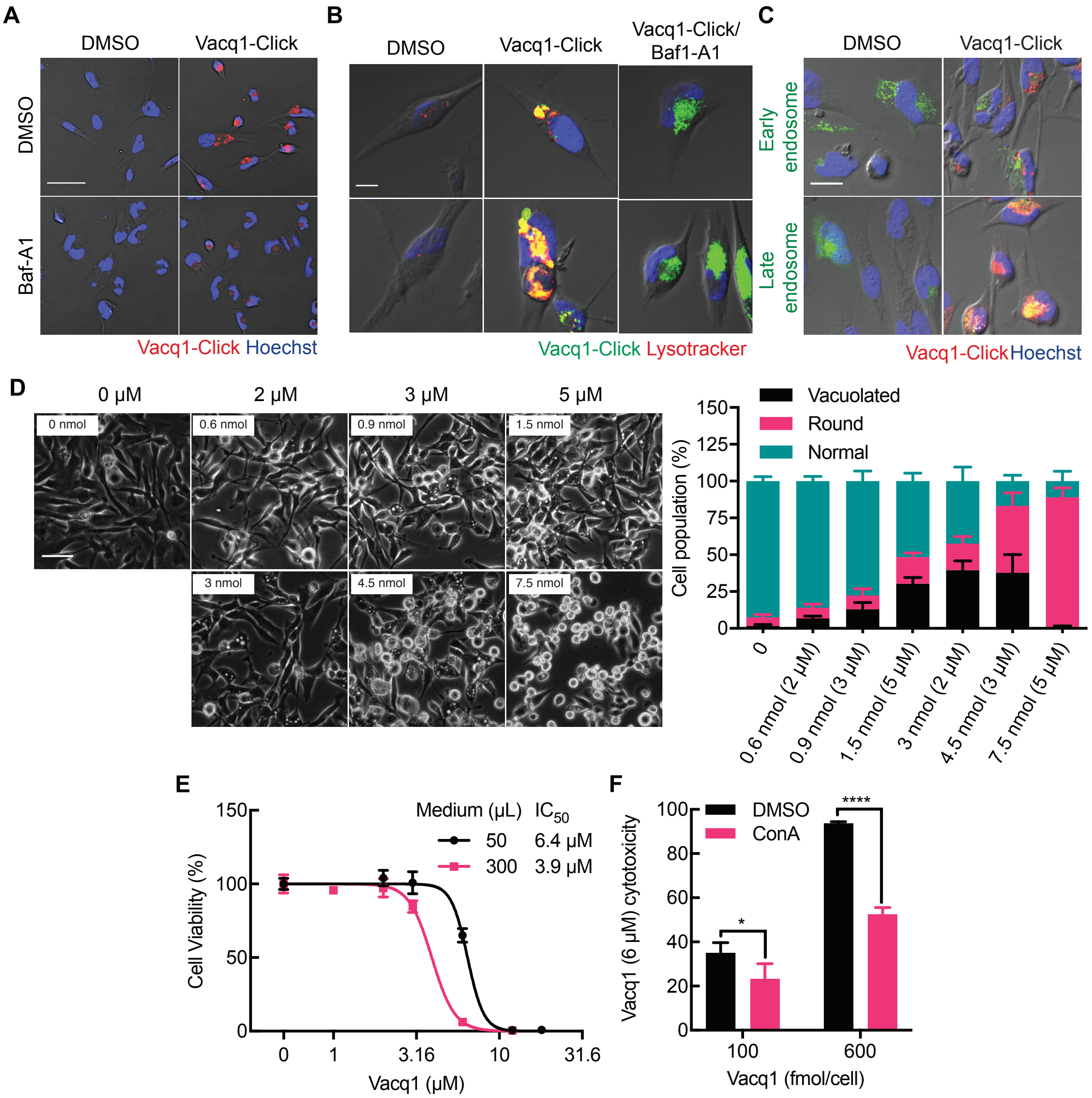

Figure S5 critical role of protonation and trapping of Vacq1 within AVOs for glioblastoma toxicity. (A) Accumulation in glioblastoma cells of functionalized Vacq1-Click in the presence of DMSO or 10 nM Baf-A1. (B) Colocalization of Vacq1-Click with AVOs in the presence of DMSO or 10 nM Baf-A1. (C) Colocalization of Vacq1-Click with early endosome or late endosome. (D) Left, representative micrographs of glioblastoma cells treated with Vacq1 for 24 h at the indicated concentrations and total quantity which was adjusted by volume change. Right, Quantification of cell morphology in each treatment condition (mean ± s.d., n = 4). (E) Cell viability of glioblastoma cells treated with increasing concentrations of Vacq1 in different volumes (mean ± s.d., n = 4). (F) Cytotoxicity of Vacq1 (6 μM) on glioblastoma cells in the presence of DMSO or 10 nM ConA (mean ± s.d., n = 4; unpaired two-tailed t-test, * P ≤ 0.05, **** P ≤ 0.0001). Scale bars: 50 μm (A, D), 10 μm (B), 20 μm (C).

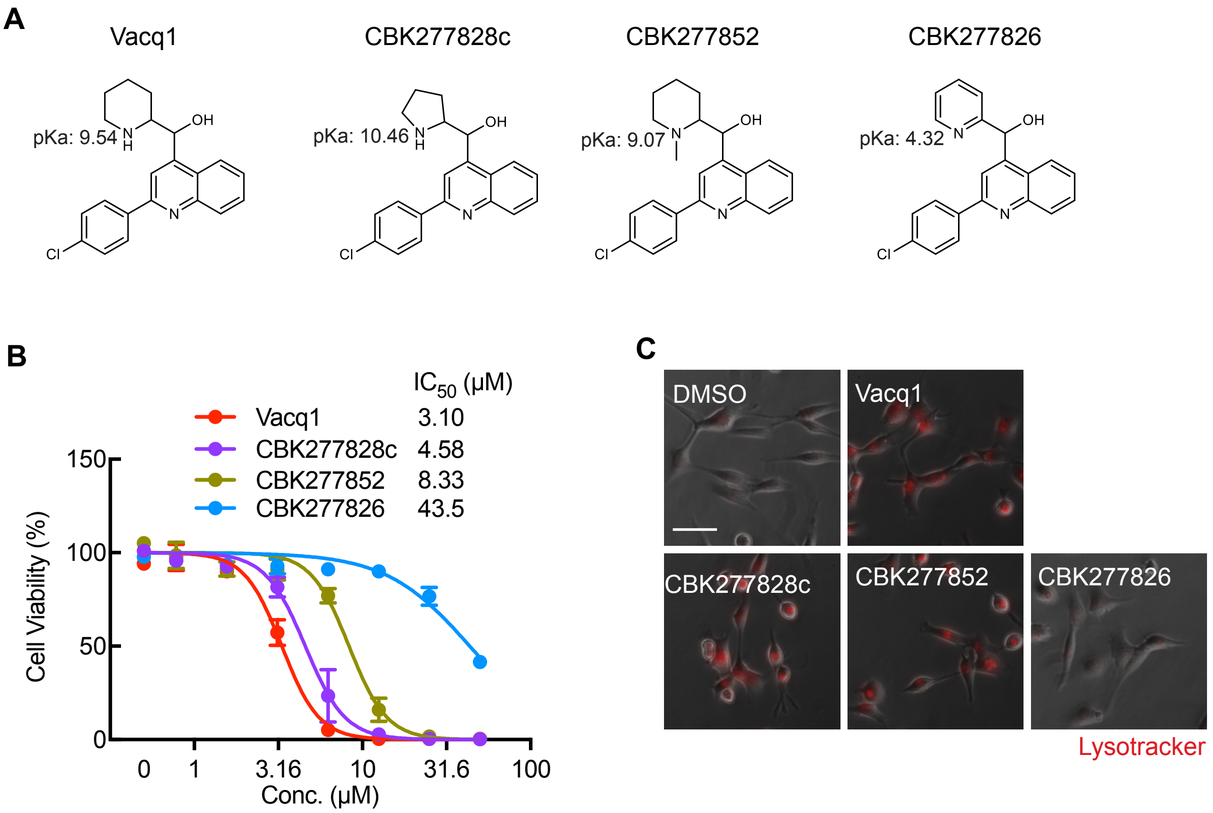

Fig. S6 Comparison of Vacq1 and its analogues on cell viability and AVO accumulation. (A) Chemical structure of Vacq1 and its analogues. (B) Cell viability of glioblastoma cells treated with Vacq1 or its analogues (mean ± s.d., n = 3). (C) AVO accumulation in glioblastoma cells treated with Vacq1 or its analogues. Scale bar: 50 μm.

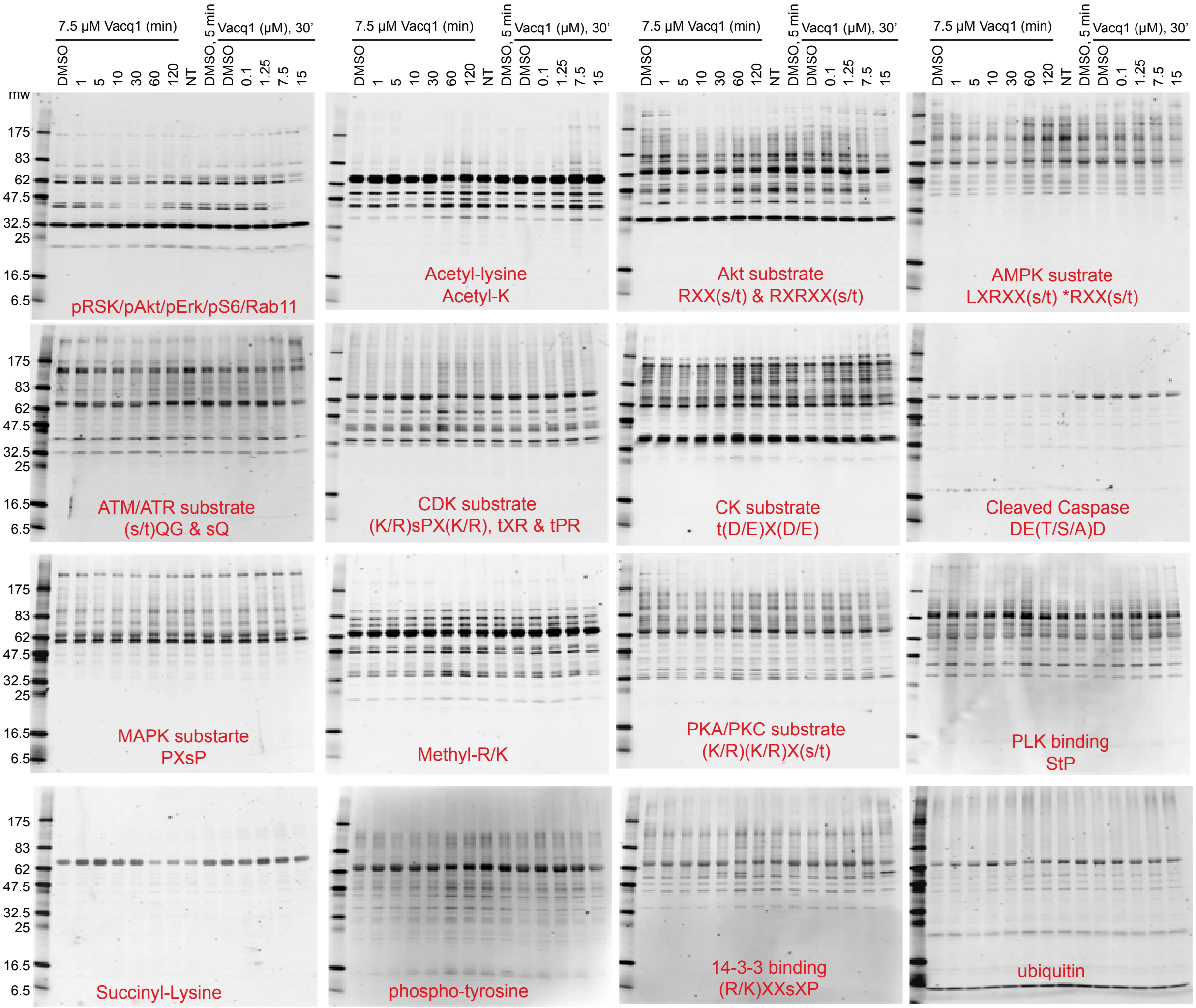

Figure S7 Profiling of signaling pathways engaged by Vacq1. KinomeView profiling of Vacq1-treated glioblastoma cells at indicated concentrations and time periods. Immunoblots were probed using a mixture of antibodies for p-RSK, p-Akt, p-Erk, pS6 and Rab11, phosphor-motif antibodies (e.g., AKT substrate with the RXX[s/t] & RXRXX(s/t) motifs), or antibodies for post-translational modification (e.g., Acetyl-Lysine), as indicated.

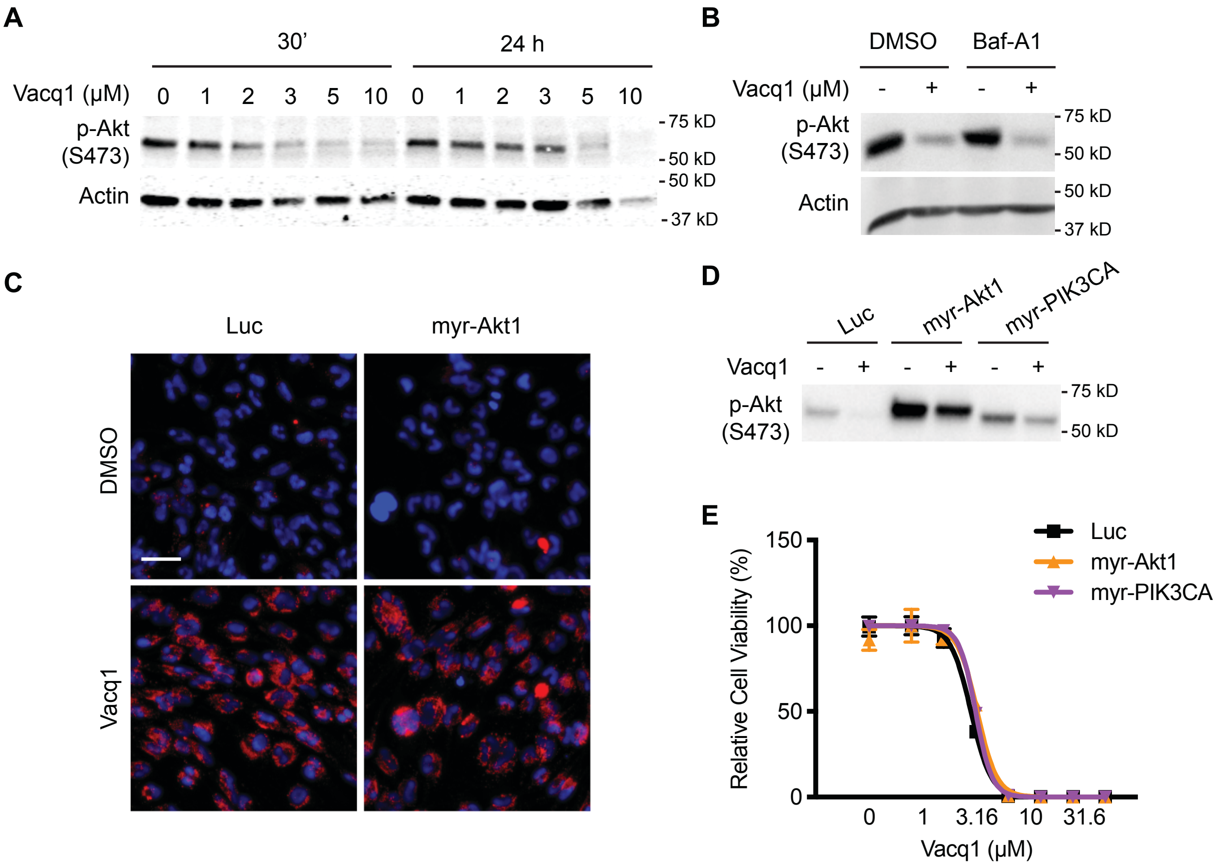

Fig. S8 Vacq1 suppression of p-Akt levels is dispensable for Vacq1-induced endolysosomal disruption and glioblastoma cell death. (A) Glioblastoma cells were treated with Vacq1 and protein from the cell lysate were analyzed for phospho-Akt (S473) at indicated times and concentrations. (B) Glioblastoma cells were treated with Vacq1 in the presence of DMSO or 10 nM Baf-A1 and protein from the cell lysates were analyzed for phospho-Akt (S473). (C) Vacq1-induced AVO accumulation in glioblastoma cells stably expressing myr-Akt1 compared to cells expressing control luciferase. (D) Immunoblots of the effects of Vacq1 on phospho-Akt (S473) in glioblastoma cells stably expressing control luciferase, myr-Akt or myr-PIK3CA. (E) Cell viability of glioblastoma cells stably expressing control luciferase, my-Akt or my-PIK3CA upon Vacq1 treatment at increasing doses (mean ± s.d., n = 3). Scale bar: 50 μm.

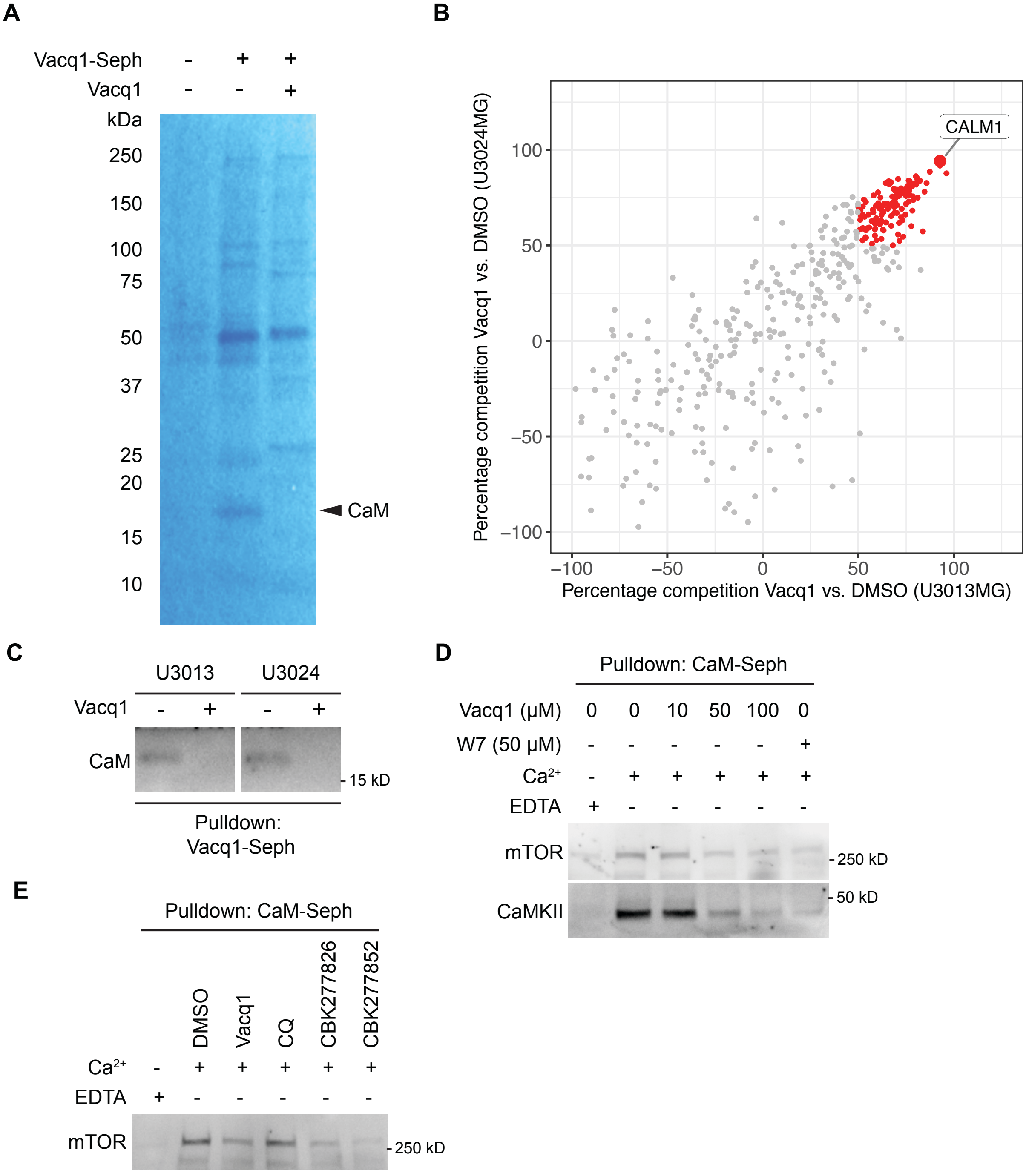

Figure S9 Chemical proteomics reveal a direct interaction of Vacq1 with calmodulin. (A) Coomassie stain from Vacq1 pull-down assay using patient derived glioblastoma U3013MG cell lysates. The Vacq1-sepharose pull-down of cell lysates were incubated with non-functionalized Vacq1 to compete non-specific interactions and demonstrate the Vacq1-specific interaction. (B) Scatter plot depicting all proteins (n = 469) identified and quantified with more than 3 unique spectra and 200 protein score in this label-free quantitative chemical proteomics experiment. Proteins are plotted as a function of the percentage competition with Vacq1 relative

to DMSO. All proteins that are specifically competed out more than 50% with Vacq1 are highlighted in red (n = 124). (C) Vacq1 pull-down and immunoblot for CaM in glioblastoma cells (U3013MG and U3024MG) with or without competition using non-functionalized Vacq1. (D) CaM-bead pull-down assay using glioblastoma cells (U3013MG) lysate in the presence of Vacq1, W7, Ca^{2+} and EDTA, as indicated. The precipitates were analyzed by immunoblotting to detect the indicated proteins. (E) CaM-bead pull-down assay using glioblastoma (U3013MG) lysate in the presence of 50 µM Vacq1, chloroquine (CQ), CBK277826 or CBK277852. The precipitates were subjected to immunoblotting to detect the indicated proteins.

Legend for Table S1

Table S1. Competition data for all 469 proteins captured on the immobilized Vacq1 (False discovery rate < 1%) with Vacq1 relative to DMSO. For each protein, the following information is provided: Protein ID, Gene name, number of unique peptides, Mascot score and % competition Vacq1 vs. DMSO.

SUPPLEMENTARY METHODS

Buffers, reagents and antibodies

Fractionation buffer is composed of 20 mM HEPES (pH 7.4), 50 mM KCl, 90 mM K-Gluconate, 1 mM EGTA, 50 mM Sucrose, and 5 mM Glucose. Cell lysis buffer is composed of 40 mM HEPES (pH 7.4), 150 mM NaCl, 2 mM EGTA, 10 mM pyrophosphate, 10 mM glycerophosphate, 1% Triton X-100 and one table of EDTA-free protease inhibitors (Roche) per 25 ml) The reagents for the organelle labeling were CellLight® Mitochondria-GFP (#C10600), CellLight® Golgi-GFP (#C10592), CellLight® ER-GFP (#C10590), CellLight® Lysosome-GFP (#C10596), CellLight® Early Endosome-GFP (#C10586) and CellLight® Late Endosome-GFP (#C10588). Primary antibodies for the immunoblot were anti-LC3B (Cell Signaling Technology, #2775), anti-p62/SQSTM1 (Cell Signaling Technology, #7695), anti-beta-actin (Abcam, #ab6276), anti-phospho Akt (S473) (Cell Signaling Technology, #4060), anti-CaM (Cell Signaling Technology, #4830), anti-mTOR (Cell Signaling Technology, #2983), anti-CAMKII (Cell Signaling Technology, #4436)

Plasmids

Luciferase-pcw107 (Addgene, #64648), myr-FLAG-Akt1-pcw107 (Addgene, #64606) or myr-FLAG-PIK3CA-pcw107 (Addgene, #64605) were a gift from David Sabatini & Kris Wood.

Cell lysis and immunoblotting

For the immunoblot analysis, U3013 cells were rinsed once with ice-cold PBS and lysed in ice-cold lysis buffer. The soluble fractions of the cell lysates were isolated by centrifugation at 15000 g for 15 min and subjected to SDS-PAGE and immunoblotting. For the immunoblotting the signal was developed by ECL Prime WB Detection Reagent (GE Healthcare) and the images were taken using Molecular Imager Chemidoc XRST (Biorad).

KinomeView profiling

U3013MG cells treated with Vacq1 was lysed in urea lysis buffer (20 mM HEPES (pH 8.0), 9 M urea, 1 mM sodium orthovanadate, 2.5 mM sodium pyrophosphate, 1 mM glycerol phosphate). The cell lysates were sonicated and centrifuged at 15000 g for 15 min. KinomeView profiling with the lysates was performed by Cell Signaling Technology.

Stable expression of myr-Akt1 and myr-PIK3CA

Lentivirus was produced by transfected HEK293FT cells with Luciferease-pcw107 (Addgene, #64648), myr-FLAG-Akt1-pcw107 (Addgene, #64606) or myr-FLAG-PIK3CA-pcw107 (Addgene, #64605) together with packaging plasmids pMD2.G and psPAX2. After 6 h, the media was changed to DMEM with 10% FBS. After 60 h, viral supernatants were harvested and centrifuged at 1500 g for 10 min to pellet cell debris. The supernatant was concentrated with Lenti-X™ Concentrator (Clontech, #631232), according to manufacturer's protocol. U3013MG cells were transduced with lentivirus-containing medium containing 4 µg/ml polybrene to establish stable expression. The medium was refreshed after 24 h and puromycin (1 µg/ml) was added for selection.

Vacq1 pull-down assay

Vacq1-sepharose was prepared through coupling reaction between NHS-activated Sepharose (Signa, #GE17-0906-01) and Vacq1 derivative (CBK278447) which the chlorophenyl moiety was replaced with benzyl(methyl)amine. U3013MG cells were lysed in ice-cold lysis buffer (40 mM HEPES, pH 7.4, 150 mM NaCl, 2 mM EGTA, 10 mM pyrophosphate, 10 mM glycerophosphate, 1% Triton X-100 and one table of EDTA-free protease inhibitors (Roche) per 25 ml). The cell debris was cleared by centrifugation at 15000 g for 15 min. The cell lysates were pre-incubated in 10 mM Vacq1. Then, Vacq1-sepharose were added and incubated with rotation for 2 h at 4ºC. The precipitates were washed three times with lysis buffer. The

precipitated proteins were denatured by adding 40 µl sample buffer and boiling for 5 minutes. The denatured proteins were resolved by 4%-20% SDS-PAGE and subjected to immunoblotting or mass spectrometry.

Mass spectrometry

Peptides that were prepared from in-gel digestion were injected onto the nLC-MS/MS system, UltiMate™ 3000 RSLCnano chromatography system and Q Exactive™ Plus Orbitrap mass spectrometer (Thermo Scientific). The peptides were separated on a homemade C18 column, 25 cm (Silica Tip 360 µm OD, 75 µm ID, New Objective, Woburn, MA, USA) with a 120 min gradient at a flow rate of 300 nl/min. The gradient went from 5-26% of buffer B (2% acetonitrile, 0.1% formic acid) in 115 min and up to 95% of buffer B in 5 min. Then, the effluent was electro sprayed into the mass spectrometer direct via the column. The survey MS spectrum was acquired at the resolution of 140,000 in the range of m/z 300-1650. MS/MS data were obtained with a higher-energy collisional dissociation (HCD) at a resolution of 17500. Quantification of the proteins was performed using the homewritten program Quanti v. 2.5.2.1. (Lyutvinskiy et al., 2013) This program performs the label-free extracted-ion-chromatogram-based quantification of peptides presented in Mascot search results considering all available isotopes and charge states. Quanti uses for quantification only reliably identified (false discover rate 0.01), first-choice, unmodified and unique sequence peptides. No fewer than two such peptides have to be present in order for a protein to be quantified. For each protein, one of the databases I.D.s was selected that covered all the identified peptide sequences for that protein. All the I.D.s corresponding to the same peptide set or subset of that peptide set.

CaM pull-down assay

U3013MG cells were lysed in ice-cold lysis buffer (40 mM HEPES (pH 7.4), 150 mM NaCl, 2 mM EGTA, 10 mM pyrophosphate, 10 mM glycerophosphate, 1% Triton X-100 and one table of EDTA-free protease inhibitors (Roche) per 25 ml). The cell debris was cleared by centrifugation at 15000 g for 15 min. The cell lysates were incubated with pre-washed CaM-sepharose (Sigma, #GE17-0529-01) with rotation for 5 h at 4ºC. The precipitates were washed three times with lysis buffer. The precipitated proteins were denatured by adding 40 µl sample buffer and boiling for 5 minutes. The denatured proteins were resolved by 4%-20% SDS-PAGE and subjected to immunoblotting.

Chemical synthesis of Vacq1 derivatives

Synthesis of Vacq1, CBK277828c, CBK277852 and CBK2778447 were previously described (Färegårdh et al., 2015). The general chemical procedures presented in this reference also apply to the preparations described here.

Preparation of CBK277826

2-(4-Chlorophenyl)-4-(pyridine-2-carbonyl)quinoline.

To a stirred solution of 2-bromopyridine (96 mg, 0.15 mmol) in dry diethyl ether (4 mL), cooled to -40 °C, *n*-butyl lithium (1.6 M in hexane, 0.94 mL, 0.15 mmol) was added, stirred for 20 min and methyl 2-(4-chlorophenyl)quinoline-4-carboxylate (150 mg, 0.50 mmol) taken in dry THF (2 mL) was added to the reaction mixture maintaining the temperature at -78 °C. The reaction was continued for 2 h (monitored by TLC). After complete consumption of the starting material, the reaction was diluted with water (10 mL) and extracted with ethyl acetate (2 x 10 mL). The combined organic extracts were washed with water (10 mL), dried over sodium sulfate, filtered, and concentrated under reduced pressure to obtain the crude material. The crude residue was purified by preparative HPLC to afford the title compound (100 mg, 58%) as an off-white solid. TLC: 10% EtOAc/ Hexanes (Rf: 0.4) ^{1}H NMR (400 MHz, $CDCl_3$): δ 8.67-8.66 (m, 1H), 8.32 (d, J = 7.5 Hz, 1H), 8.23 (d, J = 8.5 Hz, 1H), 8.11 (d, J = 8.5 Hz, 2H), 8.00 (t, J = 8.0 Hz, 1H), 7.94 (s, 1H), 7.87 (d, J = 8.0 Hz, 1H), 7.75 (t, J = 8.0 Hz, 1H), 7.74-7.26 (m, 4H). LC-MS (ESI^+): *m/z* 345 $[M+H]^+$ (96% purity).

[2-(4-chlorophenyl)quinolin-4-yl](pyridin-2-yl)methanol (CBK277826).

To a stirred solution of 2-(4-chlorophenyl)-4-(pyridine-2-carbonyl)quinoline (500 mg, 1.45 mmol) in methanol (8 mL), cooled to 0°C, sodium borohydride (109 mg, 2.90 mmol) was added and stirred for 30 min (monitored by TLC). The reaction was diluted with water (20 mL) and extracted with ethyl acetate (2 x 25 mL). The combined organic extracts were washed with water (20 mL), dried over sodium sulfate, filtered, and concentrated under reduced pressure to obtain the crude material. The crude residue was purified by silica gel column chromatography (20% ethyl acetate/hexanes) to afford **7** (200 mg, 40%) as white solid. TLC: 30% ethyl acetate / Hexanes (Rf: 0.4) ^{1}H NMR (400 MHz, $CDCl_3$): δ 8.66 (m, 1H), 7.18 (d, J = 7.6 Hz, 1H), 8.12-8.10 (m, 3H), 7.95 (s, 1H), 7.73-7.69 (m, 1H), 7.60-7.56 (m, 1H), 7.52-7.46 (m, 2H), 7.26-7.23 (m, 1H), 7.10 (d, J = 8.0 Hz, 1H), 6.49 (s, 1H), 5.49-5.47 (m, 1H). LC-MS (ESI^+): *m/z* 347 $[M+H]^+$ (99% purity).

Preparation of Vacq1-Click (CBK291469)

[4-(methylaminomethyl)phenyl]boronic acid hydrochloride

[4-[[tert-butoxycarbonyl(methyl)amino]methyl]phenyl]boronic acid (200 mg, 0.75 mmol) was suspended in 2N HCl (10 mL) and methanol (0.5 mL) was added. The reaction mixture was heated at 100°C for 15 minutes and cooled to room temperature. The reaction mixture was concentrated to give the hydrochloride as a white solid. Yield 135 mg (90%). The crude product was used as such. LC purity>90%. LC-MS (ESI^+): *m/z* 166 [M+H]+.

(R)-[2-[4-(methylaminomethyl)phenyl]-4-quinolyl]-[(2S)-1-trityl-2-piperidyl]methanol

R)-(2-bromo-4-quinolyl)-[(2S)-1-trityl-2-piperidyl]methanol (200 mg, 0.35 mmol) and potassium carbonate (aq, 2M, 0.7mL, 1.4 mmol) were dissolved in 2-methyltetrahydrofuran (5 mL) and [4-(methylaminomethyl)phenyl]boronic acid hydrochloride (107 mg, 0.53 mmol) and $PdCl_2$ (dppf) (11.6 mg, 0.01 mmol) were added. The tube was flushed with nitrogen and the reaction mixture was heated in a sealed tube at 83°C overnight. The reaction mixture was diluted with water and extracted with diethyl ether (2x50 mL). The organic phase was dried (sodium sulfate) and concentrated to give a black oil. The crude product was purified by flash chromatography on silica gel using 2% methanol in dichloromethane with 1% triethylamine as the eluent. The pure fractions were concentrated to give a brown solid. LC purity >95%. Yield 125 mg. The compound was further purified on preparative chromatography using basic buffer (NH_4HCO_3, pH 10) and acetonitrile (gradient acetonitrile 60 to 90%). The acetonitrile was removed under reduced pressure and the aqueous residue was extracted with dichloromethane (2x), dried (sodium sulfate) and evaporated to give a beige solid. Yield 70 mg (33%). LC purity>98%. LC-MS (ESI^+): *m/z* 604 [M+H]+. ^{1}H NMR (400 MHz, $CDCl_3$): δ 8.15 (m, 4H), 7.58 (m, 6H), 7.44 (m, 2H), 7.3 (m, 4H), 7.22 (m, 6H), 7.05 (m, 1H), 6.6 (m, 1H), 5.81 (m, 1H), 4.10 (m, 1H), 2.82 (s, 2H), 3.24 (m, 1H), 3.18 (br, s, 1H), 2.85 (m, 1H),2.43 (s, 3H), 2.70 (m, 1H), 1,16 (m, 2H), 0.98 (m, 2H).

3-[3-(but-3-yn-1-yl)-3H-diazirin-3-yl]-N-[(4-{4-[(R)-hydroxy(2S)-piperidin-2-ylmethyl]quinolin-2-yl}phenyl)methyl]-N-methylpropanamide (Vacq1-Click, CBK291469)

PS-EDC resin (100 mg, ca 0.05 mmol) was pre-suspended in chloroform (5 mL) and washed in a 10 mm stem block tube fitted with a screwcap. The washing with chloroform was repeated several times by inverting and centrifugation. A solution of 3-[3-(but-3-yn-1-yl)diazirin-3-yl]propanoic acid (7 mg, 0.04 mmol) and (R)-[2-[4-(methylaminomethyl)phenyl]-4-quinolyl]-

[(2S)-1-trityl-2-piperidyl]methanol (25mg, 0.04 mmol) in chloroform (5 mL) was added and the mixture was shaken on a rotary carousel for 24 hours and the reaction was monitored HPLC. Another 2 mg of the acid and 20mg of the resin was added and stirring continued for another 2h. The beads were filtered, and the remaining solution treated with diluted 5% (v/v) trifluoro acetic acid in water (0.6 mL) for 30 minutes. The solution was concentrated in vacuum and dissolved in ethyl acetate and washed withNa2CO3 (sat, aq) several times and dried over MgSO4 and concentrated in vacuum.

The crude material was dissolved in a minimal amount of 1:1 dichloromethane:diethyl ether and 1M HCl etherate added dropwise. The precipitated material was filtered into a small glass fritted filter funnel and washed with diethyl ether to give the hydrochloric salt that was dissolved in methanol and dried in vacuum. The free base was formed by treatment with ethyl acetate and sodium hydroxide aqueous solution (0.1M). The organic layer was dried over MgSO4, filtered, evaporated, and dried in vacuum to give the title compound. Yield: 10.1 mg (47%). LC purity 95%. LC-MS (ESI^+): *m/z* 510 [M+H]+. ^{1}H NMR (400 MHz, $CDCl_3$): δ 8.21 (td, J = 4.03, 7.74 Hz, 1H), 8.15 (dd, J = 7.82, 11.29 Hz, 2H), 7.96 (d, J = 8.37 Hz, 1H), 7.67 - 7.77 (m, 1H), 7.48 - 7.57 (m, 1H), 7.39 (d, J = 8.21 Hz, 1H), 7.28 - 7.33 (m, 1H), 5.46 (d, J = 3.32 Hz, 1H), 4.67 (s, 1H), 4.57 (s, 1H), 3.07 - 3.20 (m, 2H), 2.95 - 3.03 (m, 1H), 2.68 - 2.80 (m, 1H), 2.10 - 2.18 (m, 2H), 1.99 - 2.10 (m, 3H), 1.87 - 1.98 (m, 3H), 1.63 - 1.77 (m, 3H), 1.57 (d, J = 12.95 Hz, 1H), 1.29 - 1.46 (m, 3H), 1.26 (s, 1H), 1.03 - 1.21 (m, 3H)

SUPPLEMENTARY REFERENCES

Färegårdh, K., Gravenfors, Y., Ernfors, P., Hammarström, L., and Kitambi, S. (2015). Compounds and use for treating cancer, WO 2015/033228 A2.

Lyutvinskiy, Y., Yang, H., Rutishauser, D., and Zubarev, R. A. (2013). In silico instrumental response correction improves precision of label-free proteomics and accuracy of proteomics-based predictive models. Mol Cell Proteomics *12*, 2324-2331.
